# Meta-Analysis of Social Presence Effects on Stroop Task Performance

**DOI:** 10.1177/00332941241227150

**Published:** 2024-01-30

**Authors:** Teresa Garcia-Marques, Alexandre C. Fernandes

**Affiliations:** William James Center for Research, 56068 ISPA – Instituto Universitário, Lisbon, Portugal

**Keywords:** Stroop interference, presence of others, social facilitation, mere presence, attentive presence

## Abstract

In this paper, we conducted a meta-analytic review to examine the impact of social presence on individuals’ performance on the Stroop task, shedding light on the cognitive processes underlying social facilitation. We followed PRISMA guidelines to identify and include 33 relevant studies in a multivariate random-effects meta-analysis. Our results show that social presence reliably modulates Stroop interference (a measure of cognitive control); specifically, participants exhibit lower Stroop interference when performing the task in the presence of others compared to performing it in isolation. We also found that the strength of the effect varies depending on the type of social presence: it is stronger with an attentive audience compared to an inattentive one, and null with an evaluative audience. Additionally, different features of the Stroop task itself moderate the effect; the effect is stronger for the classic version of the task compared to the semantic version, and for experiments that use mixed within-block trials compared to those with homogenous blocks. We also observed a negative relationship between the number of trials and the magnitude of the effect. Overall, these findings provide insights into the mechanisms by which the presence of others affects performance on the Stroop task, and how they align with social facilitation theories.

Cognition is unlikely to remain unchanged when we are isolated from others or in the presence of others, whether it’s a mere presence or a more active type of presence. One empirical argument supporting this significant claim has been made by studies investigating the social presence effect on Stroop task performance. Considering the relevance of this claim for both our empirical studies of cognition and our social thinking environment, we aimed to assess its validity.

This paper presents a meta-analytic review of the studies that show differences in a Stroop task performed in the presence of others versus in isolation. The Stroop task (1935) is one of the most widely-used tasks in cognitive research (see MacLeod, 1991) to investigate specific cognitive mechanisms, such as control inhibition and attentional control (e.g., Laird et al., 2005; Parris et al., 2022). The task (inspired by Stroop, 1935) asks participants to name the font color of a word in a trial where the color matches the word (e.g., RED in red) or the font color mismatching the word (e.g., RED in blue). Typically, each stimulus is presented one at a time. This allows measuring the reaction time (RT) of participants, comparing them when performing the first type of trials (congruent trials) and the second type of trials (incongruent trials). The difference in RTs between Incongruent and either Congruent or Neutral trials operationalizes levels of ‘Stroop interference’ promoted by the undesirable stimuli (semantic activation).

The Stroop task is a fundamental tool in cognitive psychology that has contributed significantly to our understanding of attention, automaticity, and cognitive control ability. Although the specific processes underlying Stroop task performance are still a topic of debate (Parris et al., 2022), numerous studies have linked the task to individuals’ inhibition abilities (Miyake et al., 2000). The relevance of the Stroop task as an index of inhibition abilities has been extensively documented in meta-analyses comparing performance across different age groups (Verhaeghen & De Meersman, 1998), as well as in individuals with various disorders such as eating disorders (Dobson & Dozois, 2004), attention-deficit disorder (Lansbergen et al., 2007), and schizophrenia (Szöke et al., 2008). Stroop task are highly relevant to the field of social facilitation, as it helps to shed light on inhibition as a cognitive mechanisms that underlie differences in task performance when individuals are in the presence of others compared to when they are alone (see Huguet & Monteil, 2013 for a review). Although the initial studies by Crandall (1974), Gribbin (1974), and Lohss (1970) (see Huguet et al., 1999) suggested that the presence of others impairs Stroop task performance, the majority of papers published since MacKinnon et al. (1985) indicates that individuals perform better on the Stroop task when in the presence of others than when alone (see exceptions in Table 1- *Supplemental materials;* available at https://osf.io/n5q8p/). These various studies also investigate the relevance of the specific procedures and aspects of the Stroop task for the effect.

In this paper, we will use a meta-analytic procedure, following PRISMA guidelines, with a twofold aim: first, to determine the extent of the effect of the presence of others on Stroop performance, and second, to identify specific moderators that may shed light on the occurrence of this effect. We specifically focus on moderators that can provide valuable insights into the various explanations for social facilitation effects.

## Stroop Task and Social Facilitation

Examining one’s performance on the Stroop task both in the presence and absence of others is essential for contrasting the most relevant social facilitation theories. To comprehend the significance of this, let’s first explore the information we can glean from Stroop task performance.

Performing a Stroop task involves participants focusing on a specific feature of stimuli while disregarding another. In the classic Stroop task, this is represented by attending to the font color of a word while ignoring its meaning, such as the word ‘RED’ written in red ink. In the semantic Stroop task, participants attend to a color-associated word while ignoring the color itself, for example, the word ‘SUN’ written in red ink. All Stroop tasks have conflicting (incongruent), non-conflicting (congruent), and/or neutral trials. Although the difference in reaction times (RT) between incongruent trials and either congruent-incongruent trials or neutral-incongruent trials both serves as a measure of interference, the use of neutral trials provides a more reliable index of interference. This is because the use of congruent trials also includes facilitation due to congruence (Dalrymple-Alford, 1966; MacLeod, 1991; Parris et al., 2022).

The Stroop effect is widely recognized as one of the most robust findings in cognitive psychology, even though the field continues to grapple with the challenge of establishing a unified theoretical explanation for its underlying mechanism (Parris et al., 2022). While we won’t delve into specific mechanisms or provide an exhaustive review of alternative explanations, it’s important to recognize the various interpretations that authors may offer for specific results of the Stroop task. These interpretations help us gain insight into the cognitive processes involved in performing the task, both in social and non-social contexts.

Researchers generally agree that performance variations in the Stroop task reflect the effectiveness of our cognitive system in preventing interference, with a smaller Stroop effect indicating better efficiency. This task provides valuable insights into individuals’ inhibition or attention control abilities. However, there is ongoing controversy regarding the precise process by which interference prevention occurs. Interference can be prevented at different stages of processing, either at the early attentional level or later during conflicting response tendencies. The effects observed at the early stage primarily depend on the overlap between relevant and irrelevant stimuli, whereas those at later stages primarily involve the overlap between the irrelevant stimulus and the response tendency. It is worth noting that, although more studies tend to support the latter explanation, these perspectives are not necessarily contradictory, as the Stroop task may allow for monitoring to occur at different levels (Logan & Zbrodoff, 1998; Parris et al., 2022). A null Stroop effect indicates proficient activation of these control procedures, while a strong Stroop interference effect suggests deficiencies in initial attentional focus or later-stage interference management.

Why did researchers believe that social presence could influence performance on the Stroop task? The studies reviewed in this paper were guided by one or two well-established theories of social facilitation: Zajonc’s (1965) dominant-response account and Baron’s (1986) attentional view. These two approaches define social facilitation as a mere presence effect, suggesting that the presence of others alone is sufficient to produce an effect. However, they offer direct but contradictory predictions about how Stroop performance can be influenced by the presence of others. Zajonc’s (1965) dominant-response account assumes that social presence increases individuals’ general tendency to rely on well-learned responses. Consequently, it should lead to better performance when the correct response is dominant (a well-learned and familiar response). Since the automatic access to the ‘word meaning’ in the Stroop task is considered akin to a dominant response (Huguet et al., 1999), Zajonc’s theory predicts that the presence of others would be detrimental to Stroop performance. In contrast, Baron’s (1986) attentional account suggests that because others act as a source of distraction, individuals in this condition increase their focus on relevant information (an ‘early selection’ process) and are better at screening out irrelevant information. According to this theory, the presence of others should enhance performance in a Stroop task (Huguet et al., 1999).

However, the simplicity of these expectations is challenged by the complexity of both theories. For instance, Zajonc’s theory also proposes that the presence of others increases individuals’ arousal levels. Interestingly, research has demonstrated that heightened arousal reduces both response times and Stroop task interferences compared to non-aroused conditions (O’Malley & Poplawsky, 1971; Chajut & Algom, 2003; Callaway, 1959; Kenemans et al., 1999). On the other hand, Baron’s hypothesis regarding narrow attention is based on the assumption that others’ presence increases working memory load. Yet, research shows that working memory load has minimal impact on the magnitude of Stroop interference (Gao et al., 2007; Moss et al., 2020).

Because of the complexity of the theory assumptions, understanding the simple direction of how others’ presence impacts Stroop performance is not enough. We need to delve into the mechanisms underlying Stroop’s performance that are affected by social presence, as creatively addressed by different authors (e.g., Augustinova & Ferrand, 2012; Huguet et al., 1999; Sharma et al., 2010) and identify other likely moderators to understand the possible pathways through which this interference occurs. This is what the studies reviewed here have been about, and we specifically focus on it in this paper.

## Identifying Performance Moderators for the Meta-Analysis

A set of moderators addressed in the reviewed studies is likely to inform us about the likelihood of others’ presence effects occurring more in a later phase of processing than an earlier one. Previous studies on this set of moderators indicate that the effects of others’ presence are more likely to occur in a later phase of processing than an earlier one.

In their studies, Huguet et al. (1999) not only identified the effect of others’ presence on Stroop performance but also explored the influence of social presence on attentional processes. They examined whether participants’ worse Stroop performance in an alone condition versus the presence condition was associated with a difference in their attention to distractors. The study primarily focused on memory for Semantic Stroop words and found that participants in the presence condition did not avoid attending to the distractors. This lack of avoidance suggests that there was no interference in earlier attentional processes, pointing to social interference occurring in later attentional stages.

Further evidence is provided by Augustinova and Ferrand (2012), who compared participants’ performance in the classic Stroop task and the semantic Stroop task (Neely & Kahan, 2001) to distinguish the type of conflict affected by the presence of others (see Schmidt & Cheesman, 2005). They posited that the semantic Stroop task isolates stimulus-stimulus conflict from response-response conflict, both of which overlap in the classic Stroop task. In the classic Stroop task, the meaning of the word (e.g., REDGREEN) competes with response choices (e.g., RED and GREEN) (see Augustinova & Ferrand, 2014; Schmidt & Cheesman, 2005). Therefore, the semantic Stroop task provides a purer measure of earlier semantic activation than the classic Stroop task (see also Manwell et al., 2004; Neely & Kahan, 2001; Scaltritti et al., 2021). Their results indicated that participants performed better in the classic Stroop task than in the semantic Stroop task when others were present, suggesting that social interference occurs in later attentional processes.

Other authors have also posited the interference of social presence effects in later processing stages, albeit supported by different sets of empirical evidence. McFall et al. (2009) argue that others’ presence interferes with later Stroop-related mechanisms, as evidenced by the increased Stroop effect when response times are restricted to 1 second (e.g., Paalack et al., 1975), while the influence of others’ presence diminishes. Additionally, Sharma et al. (2010) investigated the impact of the interval between successive Stroop trials (RSI: response stimulus intervals). It is well-established that variations in RSI can influence Stroop performance (e.g., Parris et al., 2012; Rabbitt & Vyas, 1980), with shorter intervals hindering adequate preparation for each trial (Parris et al., 2012; Sharma & McKenna, 2001). If social presence interferes with earlier attentional processes, one might expect this effect to be more pronounced with shortened RSIs. However, Sharma et al. (2010) found that social presence interferes with Stroop performance even with longer RSIs.

Collectively, these studies suggest that it is crucial to examine potential differences related to the type of Stroop task, the response stimulus interval (RSI), and the response time limit provided to participants. By analyzing these factors, we aim to provide additional support for the hypothesis that interference occurs in the later stages of the process. Furthermore, we anticipate that other task-related factors may also serve as potential moderators of the effect of others’ presence on Stroop performance, helping to illuminate the possible pathways through which this interference occurs. We have strategically chosen these moderators in our approach, focusing on factors known to vary across the studies’ procedures and expected to have an impact on Stroop performance. This is the case with the number of response choices provided to participants for their responses to the Stroop task, the type of blocks, and the types of responses. Previous research shows that the number of forced response alternatives has an impact on the performance of Stroop tasks (Hasshim & Parris, 2018; Lamers et al., 2010), likely because the higher the number of alternative responses is, the more difficult the process of response selection (Cohen et al., 1998; see Kornblum et al., 1990, for a review). This impact has been explained mainly at the level of the selection of response and response conflict (Hasshim & Parris, 2018), which occurs later in processing. The type of blocks that were used in the study is also relevant because previous studies show that the pure or mixed nature of the blocks used in a Stroop task impacts participants’ performance (Ashley & Swick, 2009; Hasshim & Parris, 2018). This effect is possibly occurring either because of a response conflict (Hasshim & Parris, 2018), a carryover effect (Ashley & Swick, 2009), or because of a conflict adaptation process (Botvinick et al., 2001). And the types of responses are relevant because responding via keypress is claimed to consistently lead to smaller Stroop effects when compared to responding vocally (saying the name aloud, see Parris et al., 2022 for a review). This effect has been interpreted as informing about semantic conflict, which is significantly reduced in the keypress task (Brown & Besner, 2001; Sharma & McKenna, 1998; but see Augustinova et al., 2019). In contrast, vocal responses relate more to semantic interference, occurring at earlier stages, and less to response conflict (Sharma & McKenna, 1998). Additionally, we can argue that vocal responses in the presence of others publicly expose individuals and are likely to interfere with evaluation apprehension (Cottrell, 1968) or alertness levels (Zajonc, 1980), increasing the presence of others’ effects. Finally, because longer practice with Stroop tasks (with a greater number of trials) results in a decline in Stroop interference (Davidson et al., 2003; Dulaney & Rogers, 1994; MacLeod & Dunbar, 1988; Wilkinson & Yang, 2012), it is relevant to analyze this factor as a moderator. Practice is assumed to lead to the development of an automatic reading suppression response, or selective attention (Dulaney & Rogers, 1994), which favors earlier attentional processes.

In addition to aiding our understanding of how others’ presence impacts individuals’ Stroop performance, this meta-analysis also aims to clarify the specific type of presence that promotes this effect. Although the type of presence is a theoretically relevant factor in defining the nature of the effect of others’ presence (see Guerin, 1983, 1986, 1993), it has been largely overlooked in studies on Stroop effects. Thus, we will address the Type of Presence as a moderator of the effect (see Bond & Titus, 1983). Some theories argue that mere presence alone is insufficient to generate social facilitation effects, and the presence of inattentive individuals will not promote the effect (e.g., Cottrell’s Evaluation Apprehension Theory; Cottrell et al., 1968). Furthermore, various approaches suggest that competition or social comparison may be necessary for certain social facilitation effects (see discussions by Bond & Titus, 1983; Guerin, 1993). Consequently, the type of presence has been recognized as a crucial moderator in studying social facilitation effects.

### Method: Meta-Analytic Study

The present meta-analytic study examines the impact of social presence on individuals’ performance on the Stroop task. We employed a PRISMA procedure to select the data for analysis. For more detailed information, please refer to the Supplemental Materials available at https://osf.io/n5q8p/.

**Figure 1.**
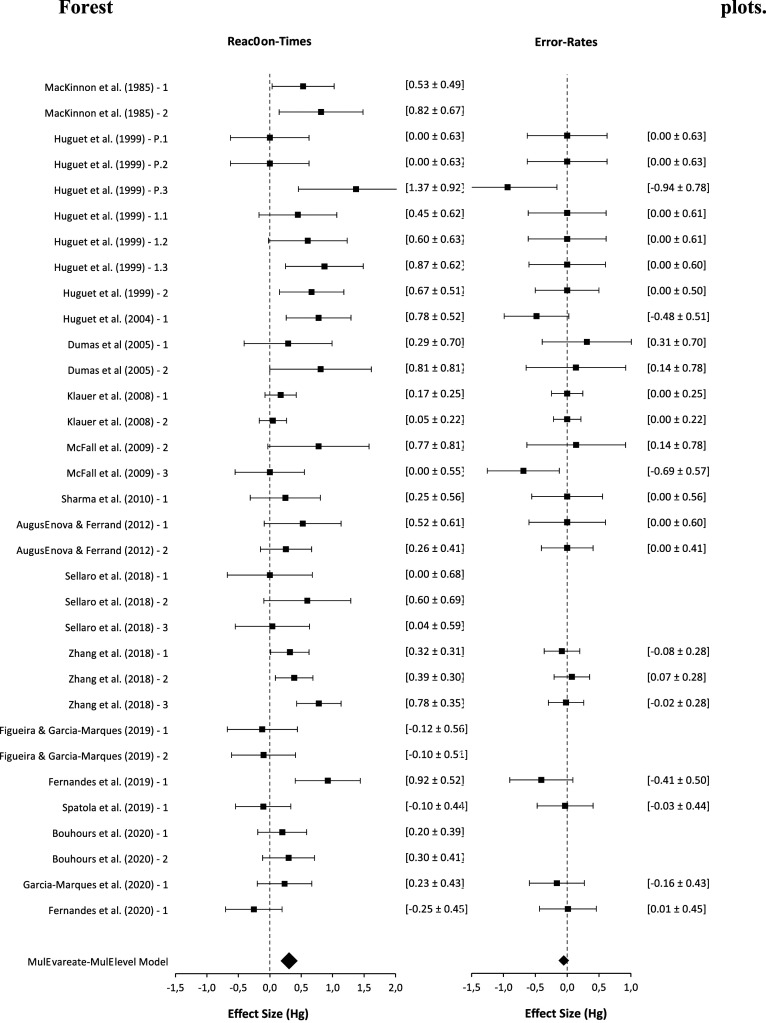
Forest plot of individual studies for bias effects of the presence of others on stroop interference.

A search combining several keywords related to the presence of others and Stroop-related keywords identified potential papers. Additional sources, including screening reference sections, conducting a descendant search on Google Scholar and Web of Knowledge, and reviewing PhD theses and conference papers (with at least an abstract in English), yielded 68 potentially relevant papers. However, to be included in the present meta-analysis, these studies had to meet specific criteria: (a) they needed to report interference effects based on reaction times; (b) they had to explicitly describe two conditions in their methodology: a ‘social condition,’ indicating the presence of others, and an ‘alone condition,’ signifying the absence of others, and (c) they needed to provide behavioral data that would enable the calculation of the Stroop effect size based on available statistical information. A total of 33 studies (from 16 papers; see Figure 1 and Supplemental materials available at https://osf.io/n5q8p/), involving 1766 participants, supported our assessment of the effects of the presence of others on Stroop interference.

The first step of this analysis evaluates the overall reliability of the effect, as well as its reliability under various forms of social presence, to examine the presence effect. The dependent measures used in this analysis include reaction times (RTs) for accurate responses, the number of errors, and the RT index of Stroop interference. Next, it investigates the role of previously identified moderators (see summary in Table 1 and details in the Supplemental Materials; https://osf.io/n5q8p/) by comparing the magnitude of the effect across different levels of these moderators.

**Table 1.** Moderators and their levels used in the analysis.

Type of presence	2 (bystanding vs. co-action) × 2 (attentive vs. inattentive)
	Incidental, evaluative, collaborative or competitive
The number of others in the experimental room	One person
	More than one person
Experimental design: Manipulation of alone and the presence of others	Within-subjects
	Between-subjects
Type of stroop task	Classic
	Semantic
	Semantic-classic Stroop, (when the tasks mix both semantic)
	Emotional Stroop
	Picture-word Stroop
Stroop control condition	Neutral (e.g., ++++_GREEN_)
	Congruent (e.g., RED_RED_)
Response stimulus interval (RSI)	Registered as a continuous factor
Blocks of trials	Mixed
	Separated
Response time limit	With time restraints
	Without time restraints
Response type	Vocal (verbal)
	Manual (keyboard)
The number of response choices (response set)	Registered as a continuous factor
The number of trials	Registered as a continuous factor
Training with neutral stimuli	Did not have a training phase
	Have a training phase
Experimental sample features	a) Credit (or payment) versus voluntary participation
	b) The age of participants was included as a continuous variable
	c) Gender of the participants, defined as the proportion of females

The analysis of the type of presence of others as a moderator was conducted using different classifications driven by its theoretical and methodological relevance. Studies were coded relying on the authors’ classification, whether the presence of others was apprehended as incidental (likely nonevaluative and nonattentive) or with other explicit goals, such as evaluative, collaborative (e.g., Sellaro et al., 2018), or competitive (e.g., Dumas et al., 2018). Studies were coded as explicitly evaluative when participants were informed that others were observing/evaluating their actions (e.g., McFall et al., 2009) or when collaborators were described as actively observing the participant (e.g., Sharma et al., 2010). In cases where the ‘presence condition’ did not match these classifications (e.g., the presence is virtual; Experiment 1, Figueira & Garcia-Marques, 2019; or implicit), they were coded accordingly.

The studies were subsequently coded differently based on the manipulated presence conditions, including a mere presence (bystanding) condition and a coaction condition where others were more or less attentive to participants’ performance (2 × 2 analysis). Initially, we coded the studies according to the authors’ classifications. For instance, a bystander study with the confederate paying attention to participants’ actions was classified as ‘mere presence,’ while a study with the experimenter absent from the room was classified as the ‘alone’ condition. Subsequently, we reclassified the studies so that ‘mere presence’ referred exclusively to situations where the presence was inattentive, and ‘alone’ conditions were only considered when the experimenter was not present in the room. This reclassification helps clarify the distinction between different experimental setups and ensures consistency in our analysis. In cases where the studies did not fit into these categories (e.g., Experiment 2 and 3, McFall et al., 2009), they were excluded from the analysis.

## Statistical Analysis and Effect Sizes

All analyses were performed using the *Metafor package* (*Version 3.2.0*, Viechtbauer, 2010) for *R* (R Core Team, 2008),^1^ following a random-effects, multilevel approach (e.g., Raudenbush & Bryk, 2002), having studies and papers as random argument of the *rma. mv* function of *Metafor*. The *restricted maximum-likelihood* (*REML*) method was used, and the effect sizes with the same level within each grouping variable received the same random effect; otherwise, effect sizes were assumed to be independent (Viechtbauer, 2010).

The effect size metric used for both outcome measures (i.e., reaction times and error rates) was Hedge’s g (Hg) with the effect sizes interpreted as small (*Hg* > 0.2), medium (*Hg* > 0.5), and large (*Hg* > 0.8). For both outcomes, a positive value in Hg indicates a lower Stroop interference value in the presence of other conditions relative to the alone condition (we avoid Cohen’s d, because it overestimates the true effect size in samples smaller than 10 participants (Borenstein, 2009; Field & Gillett, 2010); sample sizes reported in our dataset (k = 5).

We assessed heterogeneity across effect sizes by calculating a 95% confidence interval (*CI*), *Cochrane’s Q*, and *I*^*2*^ statistics indicators. Parameter estimates were obtained via *REML,* and statistical tests of model coefficients were computed via *Wald-type chi-squared tests*. Heterogeneity between the included studies is indicated by a significant statistical test for *Cochrane’s Q* (at the .05 level), and *I*^*2*^ represents the proportion of variation due to heterogeneity concerning chance (Higgins & Thompson, 2002). If high heterogeneity between studies is verified for their outcomes, the existence of moderating variables is expected, which could be subsequently tested.

Moderation was assessed through a categorical approach and a meta-regression approach. To run separate meta-analytic studies for each of the moderators, use the mods argument (rma.mv function), excluding the intercept. As the moderation test associated with Cochrane’s Q statistic for these models is relative to zero, we computed the contrasts of interest between the moderator levels. This procedure is equivalent to conducting wald-type linear combinations in a one-way ANOVA procedure. A significant effect means that between-class variance differs from the expected variance of the sampling error (Hedges & Olkin, 1985); that is, the moderator alters the effect of the presence of others on the outcome (in the case of binary moderators or between levels of moderators with 3 or more levels). For the test of multiple factors (moderators) and their interactions, we applied dummy coding to the moderators of interest (i.e., social context and attention), enabling the combination of different levels of these factors. Then, we fitted a mixed-effects meta-regression model containing the main effects of the 2 factors (mods = Factor 1 + Factor (2) and a model for the interaction between the factors (mods = Factor 1 × Factor 2). Additionally, we parameterized the model (mods = Factor 1: Factor 2) to calculate the effect of each cell of the two-way ANOVA and proceeded to the calculation of the contrasts of interest. A meta-regression procedure was used for continuous moderators based on a mixed-effects regression mode.

## Results

**Figure 2.**
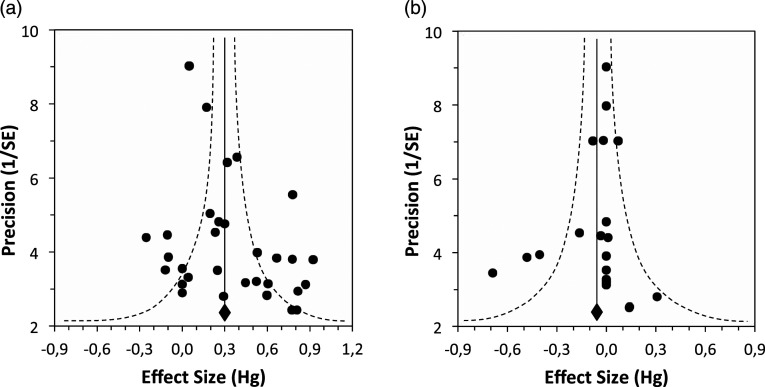
Funnel plot of precision for the overall presence of others’ effect in stroop interference, (a) for reaction times, using the random-effect model. Kendall’s tau = .241, missing studies = 5 (left), Fail-safe-N = 423. And (b) for errors using the random-effect model. Kendall’s tau = −.172, missing studies = zero (left), fail-safe-N = 0.

**Figure 3.**
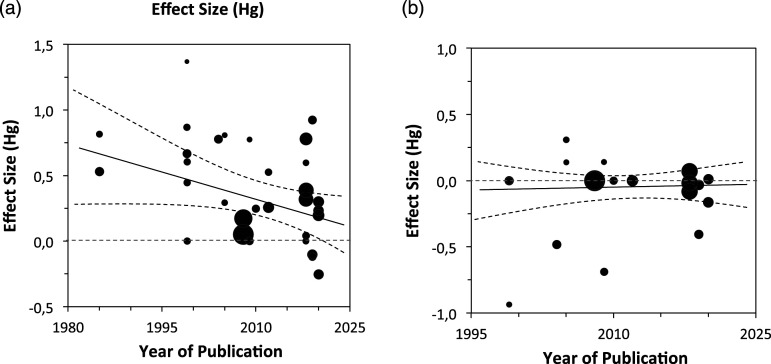
The plot portrays the nonsignificant negative change in presence effect sizes as a function of time (i.e., years) for (a) reaction times and (b) error rates the size of the circles indicates the relative contribution (random weight) of each study to the analysis.

The main results are summarized in Table 2, demonstrating that the moderation of the Stroop effect (Stroop interference) by others’ presence, when defined through reaction times (RTs), exhibits a moderate magnitude (Cohen, 1988, 1992; see Figure 2(a)). In the presence of others, the magnitude of Stroop effects is reduced, and this reduction is the focus of this meta-analysis. Importantly, most of the reviewed studies show the effect (Figure 1), and the studies’ magnitude did not change across years of publication (Figure 3(a)).

**Table 2.** Presence of Others Effects on Stroop Interference (Multivariate Meta-analysis Models).

Moderator	*k*	*g*	95% CI	*Z*_*0*_	*p*	*Q*_*W*_	*I* ^*2*^
Reaction times							
General effect	33	0.30	0.17, 0.44	4.35	<.0001	59.50**	46.03%
Excluding null effects (n.s.)	29	0.34	0.18, 0.50	4.13	<.0001	55.78**	50.25%
Errors							
General effect	24	−0.05	−0.14, 0.03	−1.24	.2145	17.91	0.00%
Excluding null effects (n.s.)	12	−0.11	−0.27, 0.04	−1.44	.1490	14.21	0.02%

+*p* < .10 *p < .05. **p < .01. ***p < .001 ****p < .0001.

### Publication Bias and Declining Effect

To assess the presence of potential publication bias, we analyzed funnel plots for both bias and error rate. These plots display observed effect sizes as a function of their precision, represented by 1/standard error (see Begg & Mazumdar, 1994). Additionally, we applied the trim-and-fill method to correct for potential publication bias, following Duval and Tweedie (2000). We also created a plot depicting the decline in effect sizes over time, highlighting the relative contribution (random weight) of each study to the analysis, and tested the significance of the slope.

#### Moderation of the Effect

##### Type of Presence: Authors’ Original Classification and Current Authors Re-Classification

Tables 3–5 provides individual effect size estimates for each type of presence category of studies. Presence categories were analyzed within a 2 (Social context) × 2 (Level of attention) factor design using ANOVA, considering both the authors’ original classification (Table 3) and our own classification (Table 4). We also present the results based on specific. classifications found in the papers (Table 5).

**Table 3.** Moderators Associated With the Manipulation and Type of Presence of Others.

Categorical Moderators	*k*	*g*	se	LL	UL	*Z*_*0*_	*p*	*Diff*	*se*_*Diff*_	*LL*_*Diff*_	*UL*_*Diff*_	*Z*_*Diff*_	*p*_*Diff*_	*Cont*
Social context × attention (ANOVA - maximum likelihood models)														
Social context factor														
Bystanding	14	0.20	0.09	0.03	0.37	2.34	.0195	−0.13	0.13	−0.38	0.13	−0.99	.3237	
Coaction	16	0.34	0.08	0.18	0.50	4.10	<.0001							
Attention factor														
Attentive	15	0.32	0.09	0.15	0.48	3.72	.0002	0.11	0.13	−0.14	0.36	0.84	.4024	
Inattentive	15	0.22	0.08	0.06	0.38	2.66	.0078							
Interaction														
Social context × attention	30	0.45	0.24	−0.02	0.91	1.87	.0616							
Parametrization (design cells)														
^1^ Bystanding-attentive	3	0.14	0.15	−0.15	0.43	0.94	.3497	−0.13	0.17	−0.46	0.21	−0.73	.4643	^1-2^
^2^ Bystanding-inattentive	11	0.26	0.09	0.09	0.44	2.99	.0028							
^3^ Coaction-attentive	12	0.50	0.09	0.33	0.66	5.82	<.0001	0.23	0.12	−0.01	0.47	1.89	.0585	^3-2^
^4^ Coaction-inattentive	4	0.18	0.14	−0.10	0.45	1.26	.2069	0.09	0.17	−0.24	0.41	0.52	.5999	^4-2^

+*p* < .10 *p < .05. **p < .01. ***p < .001 ****p < .0001.

**Table 4.** Moderators Associated With the Manipulation and Type of Presence of Others (Recategorization).

Categorical Moderators	*k*	*g*	se	LL	UL	*Z*_*0*_	*p*	*Diff*	*se*_*Diff*_	*LL*_*Diff*_	*UL*_*Diff*_	*Z*_*Diff*_	*p*_*Diff*_	*Cont*
Social context × attention (ANOVA - maximum likelihood models)														
Social context factor														
Bystanding	14	0.23	0.08	0.08	0.37	2.97	.0030	−0.13	0.11	−0.36	0.09	−1.17	.2425	
Coaction	16	0.34	0.08	0.18	0.50	4.12	<.0001							
Attention factor														
Attentive	20	0.41	0.07	0.27	0.54	6.01	<.0001	0.25	0.12	0.03	0.48	2.18	.0292	
Inattentive	10	0.16	0.09	−0.02	0.33	1.78	.0750							
Interaction														
Social context × attention	30	0.14	0.22	−0.29	0.57	0.63	.5257							
Parametrization (design cells)														
^1^ Bystanding-attentive	8	0.32	0.10	0.11	0.52	3.04	.0024	0.18	0.15	−0.11	0.47	1.22	.2229	^1-2^
^2^ Bystanding-inattentive	6	0.14	0.11	−0.07	0.35	1.26	.2061							
^3^ Coaction-attentive	12	0.50	0.09	0.33	0.66	5.77	<.0001	0.36	0.14	0.09	0.63	2.62	.0087	^3-2^
^4^ Coaction-inattentive	4	0.18	0.14	−0.10	0.45	1.27	.2030	0.04	0.18	−0.30	0.38	0.23	.8164	^4-2^

+*p* < .10 *p < .05. **p < .01. ***p < .001 ****p < .0001.

**Table 5.** Moderators Associated With the Manipulation and Type of Presence of Others.

Categorical Moderators	*k*	*g*	se	LL	UL	*Z*_*0*_	*p*	*Diff*	*se*_*Diff*_	*LL*_*Diff*_	*UL*_*Diff*_	*Z*_*Diff*_	*p*_*Diff*_	*Cont*
Aprehension of other’s goals														
^1^ Explicit evaluative	5	0.16	0.15	−0.13	0.45	1.09	.2738	−0.07	0.17	−0.40	0.26	−0.40	.6899	^1-2^
^2^ Incidental presence	16	0.23	0.09	0.06	00.4	2.64	.0082							
^3^ Explicit competition	6	0.62	0.15	0.34	0.91	4.24	<.0001	0.40	0.17	0.07	0.72	2.39	.0168	^3-2^
^4^ Explicit collaborative	6	0.38	0.14	0.10	0.66	2.68	.0074	0.15	0.17	−0.17	0.48	0.92	.3573	^4-2^
Presence manipulation (design)														
Between	25	0.33	0.08	0.17	0.49	3.94	<.0001	0.12	0.16	−0.18	0.43	0.79	.4322	
Within	8	0.21	0.14	−0.08	0.49	1.43	.1537							
Number of others (in presence condition)														
One	29	0.33	0.08	0.18	0.47	4.31	<.0001	0.12	0.18	−0.23	0.47	0.69	.4923	
More than one	4	0.20	0.16	−0.11	0.52	1.26	.2067							

+*p* < .10 *p < .05. **p < .01. ***p < .001 ****p < .0001.

**Figure 4.**
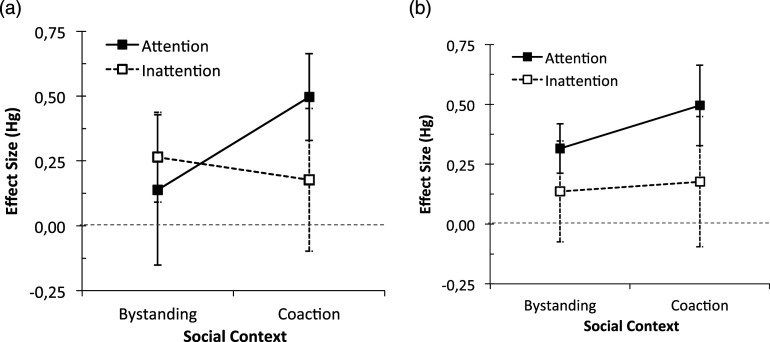
Type of presence of others (social context × attention) effects sizes (Hg) on stroop interference (with confidence intervals). (a) authors classification and (b) our classification.

The classification used in the original studies upon which the meta-analysis was based and our own classification of the studies are in agreement when it comes to concluding that the social context (bystander or coaction) does not moderate the effect. However, there is a difference in opinion regarding two other aspects: the significance of the attention main effect and the significance of the interaction component. According to the authors’ original studies classification, the interaction effect is found to be reliable (see Figure 4(a)), indicating that the effect is more pronounced in coaction-attentive conditions compared to all other three types of presence. In our classification, it is the main effect that is found to be reliable, suggesting that the effect is stronger (or possibly only detected) when others are attentive to participants’ performance than when they are not (see Figure 4(b)). Therefore, the reliability of the effect in the bystander inattentive condition (*mere presence*) is only supported by the authors’ original studies classification.

Table 5 provides detailed information about the magnitude of the effect across various dimensions of the type of presence, revealing that the dimension of evaluative apprehension is irrelevant to the effect. The analysis suggests the reliability of the effect in both competitive and cooperative settings. There is no evidence to suggest that the type of design used in the study or the number of others reliably moderates the effect of others’ presence on Stroop performance.

##### Stroop Task Features

Tables 6 and 7 summarizes the analysis of potential moderators associated with the Stroop task. Table 6 includes all types of presence, while Table 7 excludes explicit competition and cooperation manipulations to provide a clearer view of the pure effects of social facilitation^2^.

**Table 6.** Moderators Associated With the Stroop Task.

Categorical Moderators	*k*	*g*	se	LL	UL	*Z*_*0*_	*p*	*Diff*	*se*_*Diff*_	*LL*_*Diff*_	*UL*_*Diff*_	*Z*_*Diff*_	*p*_*Diff*_	*Cont*
Type of stroop task														
^1^ Classic	13	0.20	0.10	0.00	0.39	1.97	.0485							
^2^ Classic & semantic	13	0.40	0.11	0.18	0.62	3.58	.0003	0.20	0.14	−0.07	0.48	1.44	.1486	^2-1^
^3^ Semantic	5	−0.01	0.14	−0.28	0.26	−0.07	.9407	−0.21	0.14	−0.48	0.06	−1.52	.1275	^3-1^
^4^ Emotional	6	0.46	0.14	0.19	0.73	3.31	.0009	0.26	0.15	−0.03	0.56	1.76	.0779	^4-1^
Block (of control and incongruent trials)														
Separated	6	0.11	0.14	−0.16	0.39	0.79	.4276	−0.24	0.15	−0.54	0.06	−1.55	.1208	
Mixed	28	0.35	0.07	0.21	0.49	4.74	<.0001							
Time limit (to response)														
Yes (limited)	13	0.26	0.12	0.04	0.49	2.30	.0216	−0.07	0.15	−0.36	0.23	−0.43	.6649	
No (not limited)	20	0.33	0.10	0.14	0.52	3.41	.0006							
Response type														
Verbal (vocal)	9	0.36	0.15	0.07	0.64	2.45	.0144	0.07	0.17	−0.26	0.40	0.43	.6683	
Manual (keyboard)	24	0.29	0.08	0.12	0.45	3.42	.0006							
Control trials of stroop effect														
Neutral	27	0.28	0.08	0.12	0.44	3.47	.0005	−0.10	0.19	−0.47	0.27	−0.55	.5819	
Congruent	6	0.39	0.17	0.05	0.72	2.27	.0231							
Training (congruent trials)														
Yes	21	0.30	0.10	0.11	0.50	3.08	.0021	−0.02	0.17	−0.34	0.31	−0.12	.9073	
No	10	0.32	0.13	0.06	0.59	2.42	.0154							

Continuous Moderators	Mean	*k*	*Regression Weight (β)*	se	LL	UL	Z	*p*	*Intercept*	*se*_*int*_	*LL*_*int*_	*UL*_*int*_	*Z*_*int*_	*p*_*int*_
RSI (ms)	639.10	20	0.000	0.00	0.00	0.00	1.37	.1708	0.027	0.18	−0.33	0.39	0.14	.8849
Number of trials	132.42	33	−0.002	0.00	0.00	0.00	−3.38	.0007	0.561	0.09	0.39	0.74	6.30	<.0001
Number of choices	3.90	30	−0.045	0.08	−0.19	0.10	−0.60	.5482	0.487	0.30	−0.10	1.07	1.63	.1033

+*p* < .10 *p < .05. **p < .01. ***p < .001 ****p < .0001.

**Table 7.** Moderators Associated With the Stroop Task (Without Competition and Collaborative Types of Presence of Others).

Categorical Moderators	*k*	*g*	se	LL	UL	*Z*_*0*_	*p*	*Diff*	*se*_*Diff*_	*LL*_*Diff*_	*UL*_*Diff*_	*Z*_*Diff*_	*p*_*Diff*_	*Cont*
Type of stroop task														
^1^ Classic	13	0.19	0.10	0.00	0.38	1.92	.0549							
^2^ Classic & semantic	7	0.22	0.14	−0.04	0.49	1.63	.1022	0.03	0.16	−0.28	0.35	0.22	.8269	^2-1^
^3^ Semantic	5	−0.02	0.13	−0.28	0.25	−0.11	.9102	−0.20	0.14	−0.47	0.06	−1.50	.1335	^3-1^
^4^ Emotional	3	0.46	0.17	0.13	0.79	2.72	.0065	0.27	0.17	−0.06	0.60	1.59	.1108	^4-1^
Block (of control and incongruent trials)														
Separated	4	−0.08	0.14	−0.36	0.20	−0.57	.5657	−0.36	0.16	−0.67	−0.05	−2.28	.0223	
Mixed	18	0.28	0.07	0.14	0.42	3.88	<.0001							
Time limit (to response)														
Yes (limited)	7	0.20	0.14	−0.08	0.48	1.41	.1593	−0.01	0.18	−0.36	0.34	−0.05	.9618	
No (not limited)	14	0.21	0.11	0.00	0.42	1.93	.0541							
Response type														
Verbal (vocal)	4	0.31	0.20	−0.07	0.70	1.58	.1136	0.13	0.22	−0.30	0.55	0.58	.5645	
Manual (keyboard)	17	0.19	0.09	0.01	0.36	2.06	.0398							
Control trials of stroop effect														
Neutral	18	0.15	0.09	−0.03	0.32	1.61	.1067	−0.34	0.21	−0.76	0.08	−1.58	.1132	
Congruent	3	0.48	0.19	0.10	0.86	2.50	.0125							
Training (congruent trials)														
Yes	14	0.18	0.12	−0.05	0.41	1.53	.1263	−0.10	0.21	−0.50	0.31	−0.46	.6420	
No	5	0.27	0.17	−0.06	0.61	1.60	.1090							

Continuous Moderators	Mean	*k*	*Regression Weight (β)*	se	LL	UL	Z	*p*	*Intercept*	*se*_*int*_	*LL*_*int*_	*UL*_*int*_	*Z*_*int*_	*p*_*int*_
RSI (ms)	661.00	12	0.001	0.00	0.00	0.00	1.74	.0822	−0.14	0.22	−0.56	0.28	−0.65	.5152
Number of trials	168.38	21	−0.002	0.00	0.00	0.00	−2.04	.0417	0.493	0.15	0.19	0.79	3.23	.0013
Number of choices	4.05	21	−0.112	0.10	−0.30	0.08	−1.17	.2421	0.655	0.39	−0.12	1.43	1.66	.0963

+*p* < .10 *p < .05. **p < .01. ***p < .001 ****p < .0001.

**Figure 5.**
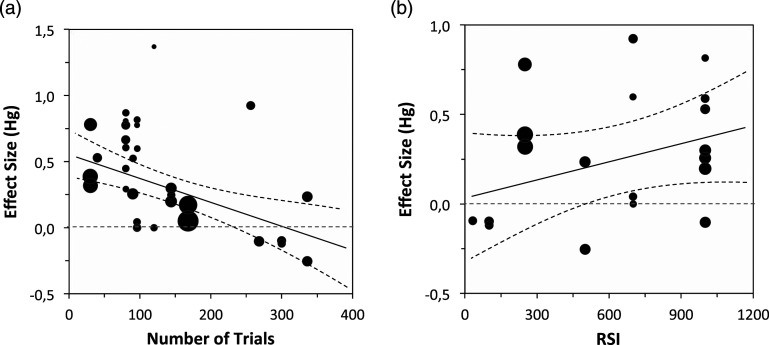
The plot represents the effect size (Hg) as a function of: (a) the number of experimental trials used in each reported study and (b) response stimulus interval (RSI) in milliseconds as reported in each study.

The analysis of Tables 6 and 7 suggests that the number of trials (also illustrated in Figure 5(a)) is a reliable moderator of the effect, with the effect being more detectable when the number of trials is lower. This could be attributed to increased practice, which leads to the development of an automatic reading suppression response, as suggested by Dulaney and Rogers (1994). This practice context facilitates earlier attentional processes and reduces the detectability of the effect. Additionally, the nature of the Stroop task is another moderator of the effect, with the effect being less reliable in a semantic task compared to a classic Stroop task. This finding supports Augustinova and Ferrand’s (2012) assumption that others’ presence effect occurs late in the process. As anticipated, the type of blocks also serves as a reliable moderator, with mixed blocks of congruent and incongruent tasks promoting stronger effects than separated blocks. The presence of other effects is most pronounced when certainty is low, requiring participants to adapt their performance trial by trial for better results.

Contrary to our initial expectation, influenced by Sharma et al. (2010), the general analysis presented in Table 6 does not support the hypothesis that the Response Stimulus Interval (RSI) is a significant moderator of the effect. To gain further clarity on this matter, we excluded the less typical social presence conditions - attentive coaction interactive presence (competition and collaboration) - from our analysis since they all employed a much longer RSI. This refined analysis yields results that align more closely with our expectations, as the RSI emerges as a significant moderator. The analysis suggests that the effect is stronger with longer RSI values (β = 0.001; *p* = .041 if unilateral; refer to Figure 5(b)) and that interactive presence likely represents a distinct process of interference with Stroop performance.

##### Experimental Features and Samples

Table 8 summarize the results testing experimental features as moderators, and show only null effects.

**Table 8.** General Moderators.

Categorical Moderators	*k*	*g*	se	LL	UL	*Z*_*0*_	*p*	*Diff*	*se*_*Diff*_	*LL*_*Diff*_	*UL*_*Diff*_	*Z*_*Diff*_	*p*_*Diff*_	*Cont*
Course credit														
Credit	12	0.29	0.12	0.06	0.52	2.49	.0128	−0.02	0.15	−0.31	0.28	−0.11	.9153	^2–1^
No credit	21	0.31	0.09	0.13	0.49	3.33	.0009							
Payment														
Yes	5	0.31	0.15	0.01	0.61	2.05	.0406	0.01	0.17	−0.33	0.35	0.06	.9530	
No	28	0.30	0.08	0.14	0.47	3.59	.0003							
Study quality														
Low	4	0.11	0.16	−0.22	0.43	0.64	.5210	−0.24	0.18	−0.6	0.11	−1.33	.1824	
High	29	0.35	0.08	0.20	0.5	4.52	<.0001							

Continuous Moderators	Mean	*k*	*Regression Weight (*β*)*	se	LL	UL	Z	*p*	*Intercept*	*se*_*int*_	*LL*_*int*_	*UL*_*int*_	*Z*_*int*_	*p*_*int*_
Year	2009	33	−0.014	0.01	−0.03	0.00	−1.99	.0467	27.914	13.88	0.71	55.12	2.01	.0444
Age (mean)	20.81	16	−0.012	0.06	−0.13	0.10	−0.20	.8441	0.473	1.23	1.95	2.89	0.38	.7018
Female percentage	76.97	29	−0.005	0.00	−0.01	0.00	−1.47	.1406	0.719	0.28	0.17	1.27	2.55	.0107

+*p* < .10 *p < .05. **p < .01. ***p < .001 ****p < .0001.

## Discussion

This meta-analysis confers validity to the claim that cognition does not remain unchanged when we are isolated from or in the presence of others. Our analysis generally supports the empirical argument that sustains this claim. In this article, we summarize researchers’ empirical arguments concerning the cognitive mechanisms underlying social facilitation effects. Our meta-analytic study reveals reliable differences in reaction times (RT) associated with Stroop performance in both social and non-social contexts (g = 0.30, [0.17; 0.44], *p* < .001). This suggests that in the presence of others, individuals are more effective at dealing with contextual interferences compared to when they are alone.

We notice that although this effect is not evident when analyzing correct responses, Stroop effects are better defined as a delay in reaction time between automatic and controlled responses. Therefore, the data support the idea that the presence of others enhances cognitive control abilities. Additionally, the non-significant decrease in effect sizes over time suggests the stability of this effect across the years of publication

An important outcome of our study is that it also reveals that not all types of presence equally facilitate the effect. This observation, consistent with findings in the social facilitation literature focusing on performance in other tasks (e.g., Bond & Titus, 1983; Guerin, 1993), is not only of practical significance but holds highly theoretical significance for two reasons.

Primarily, only mere presence and inattentive co-action are likely to represent the purest forms of the social presence factor, established as sufficient conditions for observing an impact, as proposed by Zajonc (1965). However, caution is warranted when associating mere presence with the observed effect due to our tentative stance, arising from the inability to definitively confirm the authors’ classifications of the studies. While both our classifications and those of the authors indicate that a social presence manipulation involving either a bystander or others in coaction has a similar influence on participants’ performance, our analysis suggests that the effect is stronger when others are attentive to the participant, thereby establishing an interaction. The key distinction between our classification and the authors’ lies in the discrepancy in classifying the studies as having an inattentive (mere presence) or attentive bystander. Consequently, the results do not permit a clear conclusion regarding the reliability of the effect in the mere presence condition.

The second reason why the obtained type of presence effect is of theoretical relevance is that it helps to rule out the hypothesis that an explicit evaluative goal is necessary for the effect. This result aligns with previous findings in other types of performances, as reviewed in Bond and Titus’ (1983) meta-analysis. The results challenge the notion that social facilitation relies on participants perceiving the presence of others as evaluative, as proposed by approaches such as Cottrell (1986), Geen (1981), Geen and Gange (1977), and Mullen et al. (1997). While evaluative apprehension as a specific motivational factor does not interfere with Stroop performance, it is noteworthy that other motivational factors, as explored in the literature, have also demonstrated null effects. For instance, Huguet et al. (2004) found that offering a reward to participants resulted in worse performance, and Chajut and Algom (2003) found no effect of a direct manipulation of motivation for accuracy.

Importantly, the null effects of evaluative apprehension found in this meta-analysis clarify that the significant impact observed for the ‘attentive audience’ was specifically driven by the type of presence that necessitates social interaction, such as in competition or cooperation conditions. The explanations for this effect are likely not directly accounted for by social facilitation theories, suggesting that the process by which the effect occurs may be defined by an alternative cognitive pathway than the one supporting mere presence or co-action effects. Although this is an empirical question that may be approached in the future, our analysis already offers some relevant insights for potential answers. However, we interpret these arguments with caution, as these analyses are associated with a potential loss of statistical power. As such, this remains an open question to which our meta-analytic work merely draws readers’ attention.

In summary, our analysis indicates that Stroop performance data support the claim that cognition is not the same in and out of others’ presence. However, it draws attention to the fact that there are differences in the strength of the effect based on the type of presence. It suggests that when individuals are in the presence of a bystander, it can enhance cognitive control, but this enhancement is not driven by the apprehension of being evaluated by others, as proposed by the ‘mere presence effect’ theorists (e.g., Aiello & Douthitt, 2001; Baron, 1986; see; Zajonc, 1965), which contrasts with Cottrell’s (1968) Evaluation Apprehension Theory. However, the presence of a competitive or cooperative audience may activate this effect more strongly, indicating that other social dynamics also influence cognitive control in these situations.

### Cognitive Mechanisms Underlying the Social Facilitation Effects

Our second aim in this article was to summarize researchers’ empirical arguments concerning the cognitive mechanisms underlying the social facilitation effect on cognition. In this regard, our data demonstrate, as expected based on Augustinova and Ferrand (2012), that the type of Stroop task consistently influences the effect. Specifically, the presence of others has a stronger impact on the classic Stroop task, indicating interference with later inhibitory processes at the response level. However, this empirical argument is supported only when we exclude the cooperative and competitive studies, potentially suggesting that these conditions exert their influence through a different pathway. Additionally, we did not find strong support for the empirical argument proposed by Sharma et al. (2010) based on the RSI analysis. Nonetheless, it is crucial to consider the validity of attentional interference in light of the number of trials associated with the Stroop task, which operationalizes practice. Interestingly, the number of trials emerged as a significant moderator of the effect, indicating that the presence of others is better detected initially, before the development of an automatic reading suppression response. This suggests that the differences may be driven by the fact that performance improvement is more dependent on later attentional processes, where the presence of others interferes. However, this moderation could be attributed to two alternative reasons. Firstly, it is possible that the earlier attentional processes, initiated by practice, were already enhanced in the presence of others, resulting in smaller gains. Secondly, learning may occur more rapidly in the presence of others compared to isolation conditions, as suggested by Bond and Titus’ (1983) meta-analysis. These factors contribute to the open interpretation of our conclusions.

Our results also indicate that the Stroop effect in the presence of others is moderated by the type of blocks, being more pronounced in mixed blocks conditions and not significant in separate blocks conditions. We interpret this data as evidence that the presence of others increases levels of adaptation to the expected conflict, as postulated by Botvinick et al. (2001), given that participants encounter more response conflict in mixed blocks conditions, as proposed by Hasshim and Parris (2018). The presence of others is likely facilitating the strategy with which individuals cope with higher uncertainty and imposes a trial-by-trial adaptation process (see Aben, 2017; Suh & Bugg, 2021). However, it is challenging to reconcile this finding with our data also demonstrating a greater effect for emotional stimuli. This is noteworthy, considering that previous literature has consistently reported a higher occurrence of emotional interference in block designs compared to mixed-trial dxesigns, as evidenced by a meta-analysis conducted by Phaf and Kan (2007). This suggests that the presence of others facilitates the strategy employed in mixed-trial designs, but it doesn’t independently activate the same strategies. If it did, we would expect to observe a mere dilution and cancellation of emotional interference, as discussed in studies by McKenna and Sharma (2004) and Waters et al. (2005).

Unexpectedly, some of the studied moderators yielded null effects, contradicting our initial expectations based on existing individual studies. However, this discrepancy in replication might be due to our approach of testing their roles by combining studies without considering the type of presence manipulation, which has been shown to be relevant for the effect. Future studies should explore this possibility, as our analysis lacks the statistical power to support a moderation of the moderation.

In summary, our analysis of the results leads to several preliminary conclusions concerning the cognitive mechanisms underlying the social facilitation effect on cognition. It clarifies that the impact of this social presence effect is most pronounced during the initial trials of the task. As the task complexity increases, particularly in mixed trials, the influence of social presence becomes even more prominent. These effects seem to be connected with variations in later attentional processes, as indicated by differences observed in task type and response-stimulus interval (RSI).

### Limitations and Future Directions

We refrain from providing strong conclusions about the cognitive mechanisms underlying the social facilitation effect on cognition for two main reasons. Firstly, our conclusions are based on a limited dataset, and secondly, they hinge on assumptions outlined in the introduction of this paper, supporting various authors’ empirical arguments regarding the interpretation of the studied moderations. These authors’ interpretation and assumptions may themselves be subject to criticism or debate. For instance, consider the comparison between the Classic and Semantic Stroop tasks’ performance. Our interpretation relied on arguments presented by Augustinova and Ferrand (2012). However, in a recent literature review by Parris et al. (2022) on the Stroop effect, the same author (Augustinova) challenges the informative value of comparing the Classic and Semantic Stroop tasks. According to the review, there is no clear evidence supporting a distinction between conflicting and facilitating representations at phonological, semantic, and response levels, mainly due to limitations in current measurement methods that do not allow for their isolated assessment. Additionally, recent research (Burca et al., 2021) suggests that performance in a Classic Stroop task is associated with an experience of semantic conflict. Another example to consider is our assertion in the introduction that we can rely on a comparative analysis of how participants provide their answers to infer the attentional/inhibition mechanism impacted by others’ presence. Data has shown that the Stroop effect is not stronger when using a vocal response key compared to a keyboard (Brown & Besner, 2001; Sharma & McKenna, 1998). Furthermore, the fact that interference in the performance of a Stroop task can occur at both the early and late stages of processing, not necessarily through only one pathway (e.g., Altmann & Davidson, 2001; Kornblum & Lee, 1995; Sharma & McKenna, 1998; Zhang & Kornblum, 1998), may also suggest that others’ presence may impact individuals’ performance through these two pathways.

These examples highlight that, while our analysis is grounded in the arguments of the reviewed papers, future research might propose alternative interpretations of our data. Therefore, we choose to view this analysis as providing clarification on observed effects rather than definitively establishing conclusions. Our understanding of the results may evolve with further research, incorporating various perspectives and interpretations.

### Relevance of This Data for Social Facilitation Theories

The majority of the studies we reviewed have framed the influence of others’ presence on Stroop performance within the context of social facilitation (see Belletier, Normand, & Huguet, 2019). This perspective aims to offer insights into the relative validity of two predominant theories: Zajonc’s dominant-response theory (1965) and Baron’s attentional theory (1986). However, as highlighted in the introduction of this paper, there is an ongoing debate about whether the observed Stroop modulation under the influence of others’ presence aligns more with Baron’s or Zajonc’s views.

Our data analysis aimed to tackle this question by examining the role of various moderators on the effect and comparing them to the predictions of these theories. The presence of moderators can help us comprehend the underlying mechanisms that prompt individuals to provide dominant responses or enhance their attentional control mechanisms when others are present. By investigating how these moderators interact with the effect, we gain valuable insights into which theory, either dominant-response or attentional theory, is better supported by empirical evidence. Ultimately, our data contribute to shedding light on the ongoing debate between these two theoretical perspectives.

Several independent results from this meta-analysis shed light on Zajonc’s approach. These findings include the stronger effect observed in emotional Stroop tasks compared to classic tasks, the enhanced effect in mixed block conditions versus separate blocks, and the reduced effect with increasing practice, as indicated by the number of trials. Zajonc’s theory posits that the mere presence of others influences individuals’ performance by increasing their “engagement” (as noted in studies by Blascovitz et al., 1999; Fonseca et al., 2014) and physiological arousal. Given that Stroop performance is expected to be better in arousing conditions (supported by studies like O'Malley & Poplawsky, 1971, Chajut & Algom, 2003, Callaway, 1959; Kenemans et al., 1999), this suggests that the heightened arousal experienced in the presence of others would lead to improved performance. Nonetheless, if this arousal mechanism were solely responsible for performance enhancement, one might wonder why the social presence Stroop effect is particularly pronounced in the context of an emotional Stroop task.

Moreover, according to Zajonc’s theory (1965), improved performance should only occur for simple cognitive tasks, potentially leading to impaired performance when a task becomes more complex. However, this argument is contradicted by the stronger effects observed in the mixed blocks (assumed to be more complex than homogeneous blocks), as well as by the fact that the effect is more pronounced for tasks with fewer trials, where the learning context able to simplify a task is not present, undermining the possibility that the correct response becomes a dominant response.

The results of this meta-analysis also shed light on Baron’s (1986) account. One significant insight is that the data do not support the notion that others’ presence impacts earlier attention to target stimuli, which would suggest a narrowing of participants’ attention as proposed by Baron. Another insight is that the Stroop task, performed in mixed trial blocks, shows a more pronounced effect compared to pure sequential blocks, indicating that the presence of others has a greater impact when the task itself is more complex and benefits more from narrowed attention. Additionally, the effect is more evident with a poorly learned task, a condition where Baron’s approach predicts better performance in the presence of others, in contrast to Zajonc’s theory.

The absence of unequivocal support for existing explanations of social facilitation effects, both across the literature and in the data presented in this paper, should be seen as a sign of the need for a more comprehensive theory. Such a theory is likely to be better informed by the evidence summarized in this paper and should consider more recent theories and research on cognitive control.

Various models propose that Stroop performance serves as a gauge of individuals’ ability to monitor their experiences of conflict (Botvinick et al., 2001; Cohen et al., 1990; Kalanthroff et al., 2018). Similar to Baron’s perspective, some suggest that this monitoring occurs through adjustments in attention immediately allocated to the target color on a trial-by-trial basis. For example, Botvinick et al. (2001) propose that by attending to Stroop-incongruent stimuli (detecting informational conflict), participants automatically engage in control activities. However, the mechanism facilitating increased control cannot be one of narrowing attention, as assumed by Baron, but one more closely related to Allport’s (1920) “spread of thought” hypothesis, which suggests that individuals in the presence of others attend better to contextual features and generate more context-related thoughts (Blank et al., 1976; Fonseca & Garcia-Marques, 2013; Garcia-Marques, Fernandes, Prada, et al., 2015, Garcia-Marques, Fernandes, Prada, et al., 2015; Matlin & Zajonc, 1968). The results would imply that individuals in the presence of others are more efficient at detecting the need for control. This hypothesis aligns with the fact that the effect is more clearly noticed in mixed than homogeneous trials, occurs more prominently with fewer trials, and is supported by evidence that control is likely exerted at a response level.

Another perspective is proposed by Kalanthroff et al. (2018), who rely on Braver’s et al. (2012) distinction between proactive (intentional, sustained control) and reactive control (at a response level). The authors posit that control is only exerted over automatically generated incorrect response activations (reactive control) when proactive control is weak. Zajonc’s assumption that the presence of others increases reliance on automatic responses may coincide with a reduction in the levels of proactive control. Additionally, the argument that the presence of others induces distraction could be assumed to lead to either a lower likelihood of proactive control activation in others’ presence or, as Baron assumes, an increase in proactive control. Future studies should address these possibilities, building on preliminary data offered by Belletier, Normand, Camos, et al. (2019) regarding the evaluative presence of others.

In sum, although social facilitation theories have been highly fruitful over the decades of social facilitation studies, we believe that future research should aim to understand the mechanisms underlying increased proficiency in monitoring and control in social contexts. This includes contrasting various control pathways, examining changes in proactive and reactive control, and assessing the impact on the detection of informational conflict. These hypotheses are relevant since, even by interfering at an initial level of processing, others’ presence can ultimately promote more efficient control only in later phases.

#### Conclusion

The findings of this meta-analysis are highly relevant in sustaining the claim that cognition is modulated by others’ presence. Our data consistently demonstrate the impact of others’ presence on Stroop task performance, with participants performing better in the presence of others. However, the magnitude of this effect appears to vary based on the type of social presence, raising questions about whether it is solely a ‘mere presence effect. Furthermore, the results suggest that there is currently insufficient evidence to fully understand the influence of others’ presence on cognitive control mechanisms, highlighting the need for additional theorizing and direct studies in this area. These studies should clarify why the moderation of the Stroop effect is stronger for the classic version of the task versus the semantic version, for experiments that use mixed versus homogeneous blocks, and why it decreases with the number of trials.

In sum, this meta-analytic study underscores the importance of refining the operationalization of social presence factors and contributes to both social-psychological and cognitive theories by emphasizing the role of these social factors in cognitive performance.

## Supplemental Material

Supplemental Material - Meta-Analysis of Social Presence Effects on Stroop Task Performance Supplemental Material for Meta-Analysis of Social Presence Effects on Stroop Task Performance by Teresa Garcia-Marques and Alexandre C. Fernandes in Psychological Reports.

## Data Availability

Data availability is not applicable to this article as no new data were created or analysed in this study.

## Notes

1. See details in Supplemental materials at https://osf.io/n5q8p/.

2. Competition and cooperation studies are few, not allowing the testing of the hypothesis that apparent similar results in this and another type of presence conditions occur through different attention/inhibition processes. Suggesting so, by removing these studies from the analysis we found changes in the evidence supporting RSI as a moderator and in the evidence of two other moderators: Stroop tasks (*g* = 0.22, *p* = .102; but k = 7) and Number of trials (k = 21; *β* = − 0.002; *p* = .042).

## References

[bibr1-00332941241227150] Aben B. Verguts T. Van den Bussche E. (2017). Beyond trial-by-trial adaptation: A quantification of the time scale of cognitive control. Journal of Experimental Psychology: Human Perception and Performance, 43(3), 509–517. 10.1037/xhp0000324 28080112

[bibr3-00332941241227150] Aiello J. Douthitt E. (2001). Social facilitation from Triplett to electronic performance monitoring. Group Dynamics: Theory, Research, and Practice, 5(3), 163–180. 10.1037/1089-2699.5.3.163

[bibr4-00332941241227150] Allport F. H. (1920). The influence of the group upon association and thought. Journal of Experimental Psychology, 3(3), 159–182. 10.1037/h0067891

[bibr5-00332941241227150] Altmann E. M. Davidson D. J. (2001). An integrative approach to Stroop: Combining a language model and a unified cognitive theory. Proceedings of the 23rd annual meeting of the cognitive science society (pp. 21–26): Erlbaum.

[bibr6-00332941241227150] Ashley V. Swick D. (2009). Consequences of emotional stimuli: Age differences on pure and mixed blocks of the emotional stroop. Behavioral and Brain Functions: BBF, 5(1), 1–11. 10.1186/1744-9081-5-14 19254381 PMC2661089

[bibr7-00332941241227150] * Augustinova M. Ferrand L. (2012). The influence of mere social presence on stroop interference: New evidence from the semantically-based stroop task. Journal of Experimental Social Psychology, 48(5), 1213–1216. 10.1016/j.jesp.2012.04.014

[bibr8-00332941241227150] Augustinova M. Ferrand L. (2014). Automaticity of word reading: Evidence from the semantic stroop paradigm. Current Directions in Psychological Science, 23(5), 343–348. 10.1177/0963721414540169

[bibr9-00332941241227150] Augustinova M. Silvert L. Spatola N. Ferrand L. (2018). Further investigation of distinct components of stroop interference and of their reduction by short response-stimulus intervals. Acta Psychologica, 189, 54–62. 10.1016/j.actpsy.2017.03.009 28407872

[bibr10-00332941241227150] Baron R. S. (1986). Distraction-conflict theory: Progress and problems. Advances in Experimental Social Psychology, 19(C), 1–40. 10.1016/S0065-2601(08)60211-7

[bibr11-00332941241227150] Begg C. B. Mazumdar M. (1994). Operating characteristics of a rank correlation test for publication bias. Biometrics, 50(4), 1088–1101. 10.2307/2533446 7786990

[bibr12-00332941241227150] Belletier C. Normand A. Camos V. Barrouillet P. Huguet P. (2019). Choking under experimenter’s presence: Impact on proactive control and practical consequences for psychological science. Cognition, 189(3), 60–64. 10.1016/j.cognition.2019.03.018 30927658

[bibr13-00332941241227150] Belletier C. Normand A. Huguet P. (2019). Social-Facilitation-and-Impairment effects: From motivation to cognition and the social brain. Current Directions in Psychological Science, 28(3), 260–265. 10.1177/0963721419829699

[bibr14-00332941241227150] Blank T. O. Staff I. Shaver P. (1976). Social facilitation of word associations: Further questions. Journal of Personality and Social Psychology, 34(4), 725–733. 10.1037/0022-3514.34.4.725

[bibr15-00332941241227150] Blascovich J. Mendes W. B. Hunter S. B. Salomon K. (1999). Social facilitation as challenge and threat. Journal of Personality and Social Psychology, 77(1), 68–77. 10.1037/0022-3514.77.1.68 10434409

[bibr16-00332941241227150] Bond C. F. Titus L. J. (1983). Social facilitation: A meta-analysis of 241 studies. Psychological Bulletin, 94(2), 265–292. 10.1037/0033-2909.94.2.265 6356198

[bibr17-00332941241227150] Borenstein M. Cooper H. Hedges L. Valentine J. (2009). Effect sizes for continuous data. The Handbook of Research Synthesis and Meta-Analysis, 2, 221–235. 10.7758/9781610441384

[bibr18-00332941241227150] Botvinick M. M. Braver T. S. Barch D. M. Carter C. S. Cohen J. D. (2001). Conflict monitoring and cognitive control. Psychological Review, 108(3), 624–652. 10.1037/0033-295X.108.3.624 11488380

[bibr19-00332941241227150] * Bouhours L. Camarda A. Ernst M. Osmont A. Borst G. Cassotti M. (2021). How does social evaluation influence hot and cool inhibitory control in adolescence? PLoS One, 16(9), Article e0257753. 10.1371/journal.pone.0257753 34591880 PMC8483316

[bibr20-00332941241227150] Braver T. S. (2012). The variable nature of cognitive control: A dual mechanisms framework. Trends in Cognitive Sciences, 16(2), 106–113. 10.1016/j.tics.2011.12.010 22245618 PMC3289517

[bibr21-00332941241227150] Brown M. Besner D. (2001). On a variant of Stroop’s paradigm: Which cognitions press your buttons? Memory & Cognition, 29(6), 903–904. 10.3758/BF03196419 11716063

[bibr22-00332941241227150] Burca M. Beaucousin V. Chausse P. Ferrand L. Parris B. A. Augustinova M. (2021). Is there semantic conflict in the stroop task? Experimental Psychology, 68(5), 274–283. 10.1027/1618-3169/a000530 34911356

[bibr23-00332941241227150] Callaway E. (1959). The influence of amobarbital (amylobarbitone) and methamphetamine on the focus of attention. Journal of Mental Science, 105(439), 382–392. 10.1192/bjp.105.439.382 13665299

[bibr24-00332941241227150] Chajut E. Algom D. (2003). Selective attention improves under stress: Implications for theories of social cognition. Journal of Personality and Social Psychology, 85(2), 231–248. 10.1037/0022-3514.85.2.231 12916567

[bibr25-00332941241227150] Cohen J. (1988). Statistical power analysis for the behavioral sciences (2nd ed.). Erlbaum.

[bibr26-00332941241227150] Cohen J. (1992). A power primer. Psychological Bulletin, 112(1), 155–159. 10.1037/0033-2909.112.1.155 19565683

[bibr27-00332941241227150] Cohen J. D. Dunbar K. McClelland J. L. (1990). On the control of automatic processes: A parallel distributed processing account of the stroop effect. Psychological Review, 97(3), 332–361. 10.1037/0033-295X.97.3.332 2200075

[bibr28-00332941241227150] Cohen J. D. Usher M. McClelland J. L. (1998). A PDP approach to set size effects within the stroop task: Reply to Kanne, Balota, Spieler, and Faust (1998). Psychological Review, 105(1), 188–194. 10.1037/0033-295X.105.1.188 9450376

[bibr29-00332941241227150] Core R. T. (2008). Team. R: A language and environment for statistical computing. R Foundation for Statistical. Computing.

[bibr30-00332941241227150] Cottrell N. B. (1972). Social facilitation. In McClintock C. G. (Ed.), Experimental social psychology (pp. 185–236). Holt, Rinehart and Winston.

[bibr31-00332941241227150] Cottrell N. B. Wack D. L. Sekerak G. J. Rittle R. H. (1968). Social facilitation of dominant responses by the presence of an audicence and the mere presence of others. Journal of Personality and Social Psychology, 9(3), 245–250. 10.1037/h0025902 5666972

[bibr32-00332941241227150] Crandall R. P. (1974). Influence of affective bonds on the social facilitation of multiple tasks (unpublished doctoral dissertation). University of Michigan. (University Microfilms No. 75-10, 154).

[bibr33-00332941241227150] Dalrymple-Alford E. C. Budayer B. (1966). Examination of some aspects of the Stroop color-word test. Perceptual and Motor Skills, 23(3), 1211–1214. 10.2466/pms.1966.23.3f.1211 5972923

[bibr34-00332941241227150] Davidson D. J. Zacks R. T. Williams C. C. (2003). Stroop interference, practice, and aging. Neuropsychology, Development, and Cognition. Section B, Aging, Neuropsychology and Cognition, 10(2), 85–98. 10.1076/anec.10.2.85.14463 PMC1761647 17203134

[bibr36-00332941241227150] Dobson K. S. Dozois D. J. (2004). Attentional biases in eating disorders: A meta-analytic review of stroop performance. Clinical Psychology Review, 23(8), 1001–1022. 10.1016/j.cpr.2003.09.004 14729421

[bibr37-00332941241227150] Dulaney C. L. Rogers W. A. (1994). Mechanisms underlying reduction in Stroop interference with practice for young and old adults. Journal of Experimental Psychology: Learning, Memory, and Cognition, 20(2), 470–484. 10.1037/0278-7393.20.2.470 8151280

[bibr38-00332941241227150] * Dumas F. Huguet P. Ayme E. Ayme E. (2005). Social context effects in the stroop task: When knowledge of one’s relative standing makes a difference. Current Psychology Letters, 16(2), 1–12. 10.4000/cpl.456

[bibr39-00332941241227150] Duval S. Tweedie R. (2000). Trim and fill: A simple funnel‐plot–based method of testing and adjusting for publication bias in meta-analysis. Biometrics, 56(2), 455–463. 10.1111/j.0006-341X.2000.00455.x 10877304

[bibr41-00332941241227150] * Fernandes A. C. Garcia-Marques T. (2020). The effects of presence of others in cognition: The role of executive functions. Psychonomics 2020 61st annual meeting, a virtual psychonomics experience.

[bibr42-00332941241227150] * Fernandes A. C. Garcia-Marques T. Prada M. Martins J. (2021). Emotional Interference in isolation and in others’ presence. Current Psychology, 40(12), 5783–5792. 10.1007/s12144-019-00534-0

[bibr43-00332941241227150] Field A. P. Gillett R. (2010). How to do a meta-analysis. British Journal of Mathematical and Statistical Psychology, 63(Pt 3), 665–694. 10.1348/000711010X502733 20497626

[bibr44-00332941241227150] * Figueira P. Garcia-Marques T. (2019). The other side of self-monitoring: Inhibition control in and out a social context. Análise Psicológica, 37(1), 29–39. 10.14417/ap.1498

[bibr45-00332941241227150] Figueira P. Garcia-Marques T. Fonseca R. (2012). Primação de facilitação social: Papel moderador da sensibilidade ao contexto. 7º Encontro Nacional da Associação Portuguesa de Psicologia Experimental, Lisboa, Portugal, 16 e 17 de Março de 2012.

[bibr46-00332941241227150] Fonseca R. Blascovich J. Garcia-Marques T. (2014). Challenge and threat motivation: Effects on superficial and elaborative information processing. Frontiers in Psychology, 5, 1170. 10.3389/fpsyg.2014.01170 25352823 PMC4196581

[bibr47-00332941241227150] Fonseca R. Garcia-Marques T. (2013). Back to basics: Socially facilitated situated cognition. Social Cognition, 31(2), 147–161. 10.1521/soco.2013.31.2.147

[bibr49-00332941241227150] Gao Q. Chen Z. Russell P. (2007). Working memory load and the Stroop interference effect. New Zealand Journal of Psychology, 36(3), 146–153. https://hdl.handle.net/10092/2792

[bibr50-00332941241227150] Garcia-Marques T. Fernandes A. Prada M. Fonseca R. Hagá S. (2015). Seeing the big picture: Size perception is more context sensitive in the presence of others. PLoS One, 10(11), Article e0141992. 10.1371/journal.pone.0141992 26562518 PMC4642965

[bibr51-00332941241227150] Garcia-Marques T. Fernandes A. C. Fonseca R. Prada M. (2015). Social presence and the composite face effect. Acta Psychologica, 158, 61–66. 10.1016/j.actpsy.2015.04.001 25939138

[bibr52-00332941241227150] * Garcia-Marques T. Figueira P. Fernandes A. C. (2020). Presence of others modulates socio cognitive processes. 19th general meeting of the European association of social psychology, June 30th - July 4th, 2020.

[bibr53-00332941241227150] Geen R. G. (1981). Effects of being observed on persistence at an insoluble task. British Journal of Social Psychology, 20(3), 211–216. 10.1111/j.2044-8309.1981.tb00534.x

[bibr54-00332941241227150] Geen R. G. Gange J. J. (1977). Drive theory of social facilitation: Twelve years of theory and research. Psychological Bulletin, 84(6), 1267–1288. 10.1037/0033-2909.84.6.1267

[bibr55-00332941241227150] Green R. G. (1991). Social motivation. Annual Review of Psychology, 42(1), 377–399. 10.1146/annurev.ps.42.020191.002113 2018398

[bibr56-00332941241227150] Gribbin K. J. (1974). Audience effect and age differences on tasks of increasing complexity. Dissertation Abstracts International. (Vol. 34, p. 1018B), University Microfilms. (No. 74-17, 345).

[bibr57-00332941241227150] Guerin B. (1983). Social facilitation and social monitoring: A test of three models. British Journal of Social Psychology, 22(3), 203–214. 10.1111/j.2044-8309.1983.tb00585.x

[bibr58-00332941241227150] Guerin B. (1986). Mere presence effects in humans: A review. Journal of Experimental Social Psychology, 22(1), 38–77. 10.1016/0022-1031(86)90040-5

[bibr59-00332941241227150] Guerin B. (1993). Social facilitation. Cambridge University Press.

[bibr60-00332941241227150] Hasshim N. Parris B. A. (2018). Trial type mixing substantially reduces the response set effect in the Stroop task. Acta Psychologica, 189, 43–53. 10.1016/j.actpsy.2017.03.002 28335990

[bibr61-00332941241227150] Hedges L. V. Olkin I. (1985). Statistical methods for meta-analysis. Academic Press.

[bibr63-00332941241227150] Higgins J. P. Thompson S. G. (2002). Quantifying heterogeneity in a meta‐analysis. Statistics in Medicine, 21(11), 1539–1558. 10.1002/sim.1186 12111919

[bibr64-00332941241227150] * Huguet P. Dumas F. Monteil J.-M. (2004). Competing for a desired reward in the stroop task: When attentional control is unconscious but effective versus conscious but ineffective. Canadian Journal of Experimental Psychology, 58(3), 153–167. 10.1037/h0087441 15487436

[bibr65-00332941241227150] * Huguet P. Galvaing M. P. Monteil J.-M. Dumas F. (1999). Social presence effects in the Stroop task: Further evidence for an attentional view of social facilitation. Journal of Personality and Social Psychology, 77(5), 1011–1025. 10.1037/0022-3514.77.5.1011 10573878

[bibr66-00332941241227150] Huguet P. Monteil J. M. (2013). Social context and cognitive performance: Towards a social psychology of cognition. Routledge.

[bibr68-00332941241227150] Kalanthroff E. Davelaar E. Henik A. Goldfarb L. Usher M. (2018). Task conflict and proactive control: A computational theory of the stroop task. Psychological Review, 125(1), 59–82. 10.1037/rev0000083 29035077

[bibr70-00332941241227150] Kenemans J. L. Wieleman J. S. Zeegers M. Verbaten M. N. (1999). Caffeine and stroop interference. Pharmacology, Biochemistry, and Behavior, 63(4), 589–598. 10.1016/S0091-3057(99)00022-2 10462187

[bibr71-00332941241227150] * Klauer K. C. Herfordt J. Voss A. (2008). Social presence effects on the Stroop task: Boundary conditions and an alternative account. Journal of Experimental Social Psychology, 44(2), 469–476. 10.1016/j.jesp.2007.02.009

[bibr73-00332941241227150] Kornblum S. Hasbroucq T. Osman A. (1990). Dimensional overlap: Cognitive basis for stimulus-response compatibility - a model and taxonomy. Psychological Review, 97(2), 253–270. 10.1037/0033-295X.97.2.253 2186425

[bibr74-00332941241227150] Kornblum S. Lee J.-W. (1995). Stimulus-response compatibility with relevant and irrelevant stimulus dimensions that do and do not overlap with the response. Journal of Experimental Psychology: Human Perception and Performance, 21(4), 855–875. 10.1037/0096-1523.21.4.855 7643052

[bibr75-00332941241227150] Laird A. R. McMillan K. M. Lancaster J. L. Kochunov P. Turkeltaub P. E. Pardo J. V. Fox P. T. (2005). A comparison of label‐based review and ALE meta‐analysis in the Stroop task. Human Brain Mapping, 25(1), 6–21. 10.1002/hbm.20129 15846823 PMC6871676

[bibr76-00332941241227150] Lamers M. J. Roelofs A. Rabeling-Keus I. M. (2010). Selective attention and response set in the Stroop task. Memory & Cognition, 38(7), 893–904. 10.3758/MC.38.7.893 20921102

[bibr77-00332941241227150] Lansbergen M. M. Kenemans J. L. Van Engeland H. (2007). Stroop interference and attention-deficit/hyperactivity disorder: A review and meta-analysis. Neuropsychology, 21(2), 251–262. 10.1037/0894-4105.21.2.251 17402825

[bibr80-00332941241227150] Logan G. D. Zbrodoff N. J. (1998). Stroop-type interference: Congruity effects in color naming with typewritten responses. Journal of Experimental Psychology: Human Perception and Performance, 24(3), 978–992. 10.1037/0096-1523.24.3.978

[bibr81-00332941241227150] Lohss W. E. (1970). Effects of the presence of another person in evaluative and nonevaluative roles on the performance of psychiatric patients and nonpatients (unpublished doctoral dissertation). University of Illinois. (University Microfilms No. 71-14, 849).

[bibr82-00332941241227150] * MacKinnon D. P. Geiselman R. E. Woodward J. A. (1985). The effects of effort on Stroop interference. Acta Psychologica, 58(3), 225–235. 10.1016/0001-6918(85)90022-8 3993410

[bibr83-00332941241227150] MacLeod C. M. (1991). Half a century of research on the stroop effect: An integrative review. Psychological Bulletin, 109(2), 163–203. 10.1037/0033-2909.109.2.163 2034749

[bibr84-00332941241227150] MacLeod C. M. Dunbar K. (1988). Training and stroop-like interference: Evidence for a continuum of automaticity. Journal of Experimental Psychology: Learning, Memory, and Cognition, 14(1), 126–135. 10.1037/0278-7393.14.1.126 2963892

[bibr85-00332941241227150] Manwell L. A. Roberts M. A. Besner D. (2004). Single letter coloring and spatial cuing eliminates a semantic contribution to the Stroop effect. Psychonomic Bulletin & Review, 11(3), 458–462. 10.3758/BF03196595 15376795

[bibr86-00332941241227150] Matlin M. W. Zajonc R. B. (1968). Social fa- cilitation of word associations. Journal of Personality and Social Psychology, 10(4), 455–460. 10.1037/h0026815

[bibr87-00332941241227150] * McFall S. R. Jamieson J. P. Harkins S. G. (2009). Testing the mere effort account of the evaluation-performance relationship. Journal of Personality and Social Psychology, 96(1), 135–154. 10.1037/a0012878 19210071

[bibr88-00332941241227150] McKenna F. P. Sharma D. (2004). Reversing the emotional stroop effect reveals that it is not what it seems: The role of fast and slow components. Journal of Experimental Psychology: Learning, Memory, and Cognition, 30(2), 382–392. 10.1037/0278-7393.30.2.382 14979812

[bibr89-00332941241227150] Miyake A. Friedman N. P. Emerson M. J. Witzki A. H. Howerter A. Wager T. D. (2000). The unity and diversity of executive functions and their contributions to complex “frontal lobe” tasks: A latent variable analysis. Cognitive Psychology, 41(1), 49–100. 10.1006/cogp.1999.0734 10945922

[bibr91-00332941241227150] Moss M. E. Kikumoto A. Mayr U. (2020). Does conflict resolution rely on working memory? Journal of Experimental Psychology: Learning, Memory, and Cognition, 46(12), 2410–2426. 10.1037/xlm0000801 31916832 PMC10224720

[bibr92-00332941241227150] Mullen B. Bryant B. Driskell J. E. (1997). Presence of others and arousal: An integration. Group Dynamics: Theory, Research, and Practice, 1(1), 52–64. 10.1037/1089-2699.1.1.52

[bibr93-00332941241227150] Neely J. H. Kahan T. A. (2001). Is semantic activation automatic? A critical re-evaluation. In Roediger H. L. (Ed.), The nature of remembering: Essays in honor of R. G. Crowder (pp. 69–93). American Psychological Association. 10.1037/10394-005

[bibr94-00332941241227150] O’Malley J. J. Poplawsky A. (1971). Noise-induced arousal and breadth of attention. Perceptual and Motor Skills, 33(3, Pt. 1), 887–890. 10.2466/pms.1971.33.3.887 5127209

[bibr95-00332941241227150] Pallak M. S. Pittman T. S.- Heller J. F. Munson P. (1975). The effect of arousal on Stroop color-word task performance. Bulletin of the Psychonomic Society, 6(3), 248–250. 10.3758/BF03336652

[bibr96-00332941241227150] Parris B. A. Bate S. Brown S. D. Hodgson T. L. (2012). Facilitating goal-oriented behaviour in the Stroop task: When executive control is influenced by automatic processing. PLoS One, 7(10), Article e46994. 10.1371/journal.pone.0046994 23056553 PMC3466271

[bibr97-00332941241227150] Parris B. A. Hasshim N. Wadsley M. Augustinova M. Ferrand L. (2022). The loci of stroop effects: A critical review of methods and evidence for levels of processing contributing to color-word stroop effects and the implications for the loci of attentional selection. Psychological Research, 86(4), 1029–1053. 10.1007/s00426-021-01554-x 34389901 PMC9090875

[bibr98-00332941241227150] Phaf R. H. Kan K. J. (2007). The automaticity of emotional stroop: A meta-analysis. Journal of Behavior Therapy and Experimental Psychiatry, 38(2), 184–199. 10.1016/j.jbtep.2006.10.008 17112461

[bibr99-00332941241227150] Rabbitt P. Vyas S. (1980). Actively controlling anticipation of irregular events. Quarterly Journal of Experimental Psychology, 32(3), 435–446. 10.1080/14640748008401837

[bibr100-00332941241227150] Raudenbush S. W. Bryk A. S. (2002). Hierarchical linear models. Applications and data analysis methods (2nd ed.). Sage Publications.

[bibr102-00332941241227150] Scaltritti M. Job R. Sulpizio S. (2022). Different types of semantic interference, same lapses of attention: Evidence from Stroop tasks. Memory & Cognition, 50(5), 898–910. 10.3758/s13421-021-01256-0 35040025

[bibr103-00332941241227150] Schmidt J. R. Cheesman J. (2005). Dissociating stimulus-stimulus and response-response effects in the Stroop task. Canadian Journal of Experimental Psychology/Revue Canadienne de Psychologie Expérimentale, 59(2), 132–138. 10.1037/h0087468 16035346

[bibr105-00332941241227150] * Sellaro R. Treccani B. Cubelli R. (2020). When task sharing reduces interference: Evidence for division-of-labour in stroop-like tasks. Psychological Research, 84(2), 327–342. 10.1007/s00426-018-1044-1 29971545

[bibr107-00332941241227150] * Sharma D. Booth R. Brown R. Huguet P. (2010). Exploring the temporal dynamics of social facilitation in the Stroop task. Psychonomic Bulletin & Review, 17(1), 52–58. 10.3758/PBR.17.1.52 20081161

[bibr108-00332941241227150] Sharma D. McKenna F. P. (1998). Differential components of the manual and vocal Stroop tasks. Memory & Cognition, 26(5), 1033–1040. 10.3758/BF03201181 9796234

[bibr109-00332941241227150] Sharma D. McKenna F. P. (2001). The role of time pressure on the emotional Stroop task. British Journal of Psychology, 92(3), 471–481. 10.1348/000712601162293 11802885

[bibr110-00332941241227150] * Spatola N. Belletier C. Chausse P. Augustinova M. Normand A. Barra V. Ferrand L. Huguet P. Huguet P. (2019). Improved cognitive control in presence of anthropomorphized robots. International Journal of Social Robotics, 11(3), 463–476. 10.1007/s12369-018-00511-w

[bibr112-00332941241227150] Stroop J. R. (1935). Studies of interference in serial verbal reactions. Journal of Experimental Psychology, 18(6), 643–662. 10.1037/h0054651

[bibr113-00332941241227150] Suh J. Bugg J. M. (2021). The shaping of cognitive control based on the adaptive weighting of expectations and experience. Journal of Experimental Psychology: Learning, Memory, and Cognition, 47(10), 1563–1584. 10.1037/xlm0001056 34570546 PMC8758525

[bibr114-00332941241227150] Szöke A. Trandafir A. Dupont M. E. Meary A. Schürhoff F. Leboyer M. (2008). Longitudinal studies of cognition in schizophrenia: meta-analysis. The British Journal of Psychiatry: The Journal of Mental Science, 192(4), 248–257. 10.1192/bjp.bp.106.029009 18378982

[bibr117-00332941241227150] Verhaeghen P. De Meersman L. (1998). Aging and the stroop effect: A meta-analysis. Psychology and Aging, 13(1), 120–126. 10.1037/0882-7974.13.1.120 9533194

[bibr118-00332941241227150] Viechtbauer W. (2010). Conducting meta-analyses in R with the metafor package. Journal of Statistical Software, 36(3), 1–48. 10.18637/jss.v036.i03

[bibr120-00332941241227150] Waters A. J. Sayette M. A. Franken I. H. Schwartz J. E. (2005). Generalizability of carry-over effects in the emotional Stroop task. Behaviour Research and Therapy, 43(6), 715–732. 10.1016/j.brat.2004.06.003 15890165

[bibr121-00332941241227150] Wilkinson A. J. Yang L. (2012). Plasticity of inhibition in older adults: Retest practice and transfer effects. Psychology and Aging, 27(3), 606–615. 10.1037/a0025926 22182362

[bibr123-00332941241227150] Yamaguchi M. Clarke E. L. Egan D. L. (2018). Is your color my color? Dividing the labor of the stroop task between co-actors. Frontiers in Psychology, 9, 1407. 10.3389/fpsyg.2018.01407 30131747 PMC6090138

[bibr124-00332941241227150] Zajonc R. B. (1965). Social facilitation. Science, 149(3681), 269–274. 10.1126/science.149.3681.269 14300526

[bibr125-00332941241227150] Zajonc R. B. (1980). Compresence. In Paulus P. B. (Ed.), Psychology of group influence. Erlbaum.

[bibr126-00332941241227150] Zhang H. Kornblum S. (1998). The effects of stimulus–response mapping and irrelevant stimulus–response and stimulus–stimulus overlap in four-choice Stroop tasks with single-carrier stimuli. Journal of Experimental Psychology: Human Perception and Performance, 24(1), 3–19. 10.1037//0096-1523.24.1.3 9483821

[bibr127-00332941241227150] * Zhang H. Zhang X. Liu X. Yang H. Shi J. (2020). Inhibitory process of collaborative inhibition: Assessment using an emotional stroop task. Psychological Reports, 123(2), 300–324. 10.1177/0033294118805007 30428267

