# Supplementary material for: Meta-Analysis of Social Presence Effects on Stroop Task Performance
Source: Psychol Rep. 2024 Jan 30;129(1):29–62. doi: 10.1177/00332941241227150 (PMC12717298; doi:10.1177/00332941241227150)
Supplement: Supplemental Material - Meta-Analysis of Social Presence Effects on Stroop Task Performance [file sj-pdf-1-prx-10.1177_00332941241227150.pdf]

Supplemental material

For the paper: Garcia-Marques, T. & Fernandes, A.: **Meta-analysis of Social Presence Effects on Stroop Task Performance**

Index:

1. ***PRISMA guidelines followed for this meta-analysis***
2. ***Coding for Moderators***
3. **Data Extraction and Computation of Effect Sizes**
4. **Meta-analytic Procedures**

1,PRISMA guidelines followed for this meta-analysis

Search Strategies and Study Selection

Multiple search strategies were employed to identify studies in the literature that provide data on the effects of the presence of others on interference indices in Stroop tasks. No previous reviews have cited studies on the effects of the presence of others in Stroop interference before 1968. Thus, the publication search was limited from this date to the present year. The search was completed in June 2021 using keywords identified in previous review articles (e.g., Belletier et al., 2019; Bond & Titus, 1983; Eastvold et al., 2012; Guerin, 1986; Huguet et al., 1999; Steinmetz & Pfattheicher, 2017; Seitchik et al., 2017) and performed in the following databases: *PsycInfo*, *Scopus*, *PubMed*, *Web of Knowledge* and *Google Scholar*.

The search combined several *other keywords* (presence of others, audience, alone, observer, bystander, co-actor, confederate) with specific *social context keywords* (social comparison, social facilitation, evaluation, competition, collaboration, mere presence, coaction) and *Stroop keywords* (Stroop task, color recognition task, interference, inhibition, incongruent trials) (see *Appendix A* for a complete set of combinations). A total of 8,548 potential papers were selected following this initial search procedure (see *Figure 2*).

Furthermore, we screened the reference section of all eligible articles and related reviews and performed a descendant search in *Google Scholar* and *Web of Knowledge* to identify additional papers. Studies presented in different Ph.D. theses (e.g., Crandall, 1975; Gribbin, 1974; Lindman, 2004; Lohss, 1971). conference papers (e.g., Agrigoroaie & Tapus, 2017; Fernandes & Garcia-Marques, 2020; Figueira et al., 2012; Garcia-Marques et al., 2020; Sharma et al., 2005), and all non-English articles

that have at least the abstract in English were considered for inclusion. Due to the lack or eventual deficiencies in the review processes or methodologies of these publications, the type of publication was defined as a moderator. This strategy should reduce a potential file-drawer effect (Borenstein et al., 2009; Franco et al., 2014; Lishner, 2021). Following this second step, an additional 68 papers were found, excluding replicates selected in the database-screening step (see *Figure 1*).

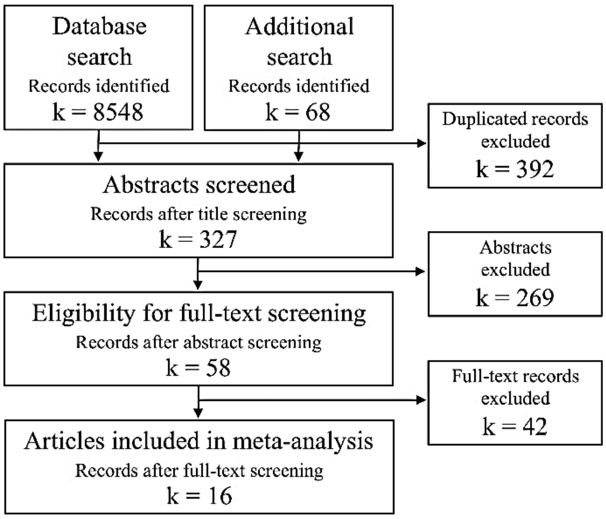

Figure 1. PRISMA flowchart summarizing the sequential, step-by-step processes involved in the literature search, screening, and article selection to the present meta-analysis (applying inclusion and exclusion criteria).

Inclusion and Exclusion Criteria

To be included in the present meta-analysis, a study had to report interference effects based either on reaction times or error rates, using any variant of the classical Stroop tasks (Stroop, 1935), and its moderation by the presence of others.

Studies were considered for inclusion if they reported a measure of interference, computed as a difference between averaged reaction times (or error rates) in incongruent and control trials (e.g., Augustinova & Ferrand, 2012; Figueira

& Garcia-Marques, 2019; MacKinnon et al., 1985), or statistically addressed an effect based on a comparison between incongruent and control trials (e.g., Dumas et al., 2005; Huguet et al., 1999; McFall et al., 2009). Studies that refer only to the effect of the presence of others in incongruent trials were excluded (e.g., Saunders et al., 2017; Yamaguchi et al., 2018) since it is not possible to dissociate effects. Studies that reported only the total time to complete the Stroop task (or different trial blocks) were also excluded (e.g., Gribbin, 1974; Kehrer et al., 2000; Timaeus & Lueck, 1968) because they do not target Stroop effects. Studies that presented only implicit measures such as those associated with physiological responses were also excluded (e.g., Demiral et al., 2016).

The main meta-analysis included studies that unequivocally reported in their method the presence of two other conditions: a social condition (i.e., in the presence of others) and a condition of being alone (i.e., without the presence of others). We excluded cases in which the alone condition was defined differently, either as the absence of other participants but maintaining the presence of the experimenter in the same room (e.g., Kehrer et al., 2000; Lindman, 2004) or as the presence of a nonactive participant (e.g., Saunders et al., 2017; Yamaguchi et al., 2018). We also excluded cases in which there were doubts about the use of this criterion (e.g., Agrigoroaie & Tapus, 2017; Demiral et al., 2016).

Studies must report behavioral data in the article or supplemental materials—*reaction times or error rates as dependent variables*—that would allow the calculation of the Stroop effect size from statistical information.

After the eligibility analysis, a total of 33 studies (from 16 papers, with data from 1,766 participants) supported our assessment of the presence of others' effects on Stroop interference.

1. Coding for Moderators

The potentially moderating variables available from the articles reporting eligible studies were coded for meta-analysis. These included variables associated with the type of presence of others, variables associated with the Stroop task features, and variables defined by the experimental samples used.

Type of presence

To approach the type of presence as a moderator we followed two strategies: 1) by aggregating studies into the four types of presence, that crossed two relevant dimensions, social context and attention (see Green, 1991; Guerin, 1983) and 2) by isolating dimensions regarding how participants a*pprehend the presence of the other*, the *number of others* present, and the type of manipulation done, *within or between* participants.

The two crossed factors s*ocial context (*bystander versus coaction) *and attention* (attentive versus inattentive) were first supported in the authors' own categorization of their experimental conditions. However, to have more objective comparison we run our own classification defining: a) a bystander manipulation when the presence is passive and others were not engaged in the performance of the same task that the participant was performing (e.g., Bouhours et al., 2020; Sharma et al., 2010) and a coaction manipulation when others are engaged in the same experimental task that the participant (e.g., Dumas et al., 2005; Fernandes et al., 2019) and b) an a*ttention* manipulation when an audience at some moment was observing the participant during his/her performance (e.g., Augustinova & Ferrand, 2012) and an *inattentive* manipulation when others' presence was incidental, invisible (e.g., Huguet

et al., 1999), or engaged in a task that did not allow them to attend to observe participants (e.g., Klauer et al., 2008), and the context carefully prevented social comparisons (e.g., Fernandes et al., 2019). An inattentive bystander operationalized the mere presence condition (Guerin, 1986; Zajonc, 1965) and an attentive bystander is a typical evaluative setting. An attentive coaction was considered if others' performance was defined as relevant for the participants (either for the goal of competition or of collaboration) if they were explicitly informed that their performance was being evaluated (e.g., McFall et al., 2009), or if they were in a social comparison context (e.g., Huguet et al., 1999). In the cases where the "presence condition" did not match these classifications (e.g., the presence is virtual; *exp.1*, Figueira & Garcia-Marques, 2019; or an implicit, *exp. 2 and 3*, McFall et al., 2009), the studies were ignored in this specific analysis.

Our criteria did not converge with the authors' own classification, especially concerning mere presence. Some studies (e.g., Augustinova & Ferrand, 2012; Huguet et al., 1999; Sharma et al., 2010) classified as a mere presence condition (for us an inattentive bystander) a condition where the participant's performance was observed 60–70% of the time, with the observer engaged in a no relevant task (for us, an attentive co-actor). As such, we considered the two classifications in our analysis, see Table 1 (see legend).

Table 1

Characteristics of Studies Included in the Meta-Analysis

Experiment		Sample				Presence of Others						Stroop Task								
Study	Exp	N	Credit	Age	Sex	Design	NOth	Context	Attention	Aa	Other's Goal	Type	Control	RSI	Block	Time	Response	Choice	Trials	Pratice
MacKinnon et al. (1985)	1	64	Yes		50	B	1	Coaction	Attentive	A	Competition	Classic & Semantic	Nt		Separated	No	Verbal	4	40	No
MacKinnon et al. (1985)	2	36	Yes		50	B	1	Coaction	Attentive	A	Competition	Classic & Semantic	Nt		Separated	No	Verbal	4	96	No
Huguet et al. (1999)	P [1]	8	No		100	W	1	Bystanding	Inattentive	I	Incidental	Classic	Nt		Mixed	No	Keyboard	4	120	Yes
Huguet et al. (1999)	P [2]	8	No		100	W	1	Bystanding	Inattentive	I	Incidental	Classic	Nt		Mixed	No	Keyboard	4	120	Yes
Huguet et al. (1999)	P [3]	8	No		100	W	1	Bystanding	Attentive	I	Incidental	Classic	Nt		Mixed	No	Keyboard	4	120	Yes
Huguet et al. (1999)	1 [1]	40	No		56	B	1	Bystanding	Inattentive	I	Incidental	Classic & Semantic	Nt		Mixed	No	Keyboard	4	80	Yes
Huguet et al. (1999)	1 [2]	40	No		56	B	1	Bystanding	Inattentive	I	Incidental	Classic & Semantic	Nt		Mixed	No	Keyboard	4	80	Yes
Huguet et al. (1999)	1 [3]	42	No		56	B	1	Bystanding	Attentive	I	Incidental	Classic & Semantic	Nt		Mixed	No	Keyboard	4	80	Yes
Huguet et al. (1999)	2	80	No		50	B	1	Coaction	Attentive	A	Competition	Classic & Semantic	Nt		Mixed	No	Keyboard	4	80	Yes
Huguet et al. (2004)	1	80	Yes		100	B	1	Coaction	Attentive	A	Competition	Classic & Semantic	Nt		Mixed	No	Keyboard	4	80	Yes
Dumas et al. (2005)	1	42	Yes	21	100	B	1	Coaction	Attentive	A	Competition	Classic & Semantic	Nt	1000	Mixed	No	Keyboard	4	80	Yes
Dumas et al. (2005)	2	32	Yes	20	81	B	1	Coaction	Attentive	A	Competition	Classic & Semantic	Nt	1000	Mixed	No	Keyboard	5	80	Yes
Klauer et al. (2008)	1	62	Paid			W	1	Bystanding	Inattentive	I	Incidental	Classic & Semantic	Nt		Mixed	No	Keyboard	4	168	Yes
Klauer et al. (2008)	2	80	Paid			W	1	Bystanding	Inattentive	I	Incidental	Classic & Semantic	Nt		Mixed	No	Keyboard	4	168	Yes
McFall et al. (2009)	2	24	Yes		50	B		Other			Evaluative	Classic	Nt		Mixed	Yes	Verbal	4	96	No
McFall et al. (2009)	3	49	Yes		51	B		Other			Evaluative	Classic	Nt		Mixed	Yes	Verbal	5	96	No
Sharma et al. (2010)	1	48	Paid		50	B	1	Bystanding	Attentive	I	Incidental	Classic	Nt	32-1000	Mixed	No	Keyboard	4	144	Yes
Augustinova & Ferrand (2012)	1	41	Yes			B	1	Bystanding	Attentive	I	Incidental	Classic & Semantic	Nt	1000	Mixed	Yes	Verbal	6	90	Yes
Augustinova & Ferrand (2012)	2	92	Yes			B	1	Bystanding	Attentive	I	Incidental	Classic & Semantic	Nt	1000	Mixed	Yes	Verbal	6	90	Yes
Sellaro et al. (2018)	1	32	No	24	91	B	1	Coaction	Attentive	A	Collaborative	Picture-Word	Cg	700	Mixed	Yes	Verbal		96	No

(Table 1 continuation)

Sellaro et al. (2018)	2	32	No	24	91	B	1	Coaction	Attentive	A	Collaborative	Picture-Word	Cg	700	Mixed	Yes	Verbal		96	No
Sellaro et al. (2018)	3	48	No	24	90	B	1	Coaction	Attentive	A	Collaborative	Picture-Word	Cg	700	Mixed	Yes	Verbal		96	No
Zhang et al. (2018)	1	48	No	21	100	W	1	Coaction	Attentive	A	Collaborative	Emotional Stroop	Nt	250	Mixed	Yes	Keyboard	2	30	Yes
Zhang et al. (2018)	2	48	No	21	100	W	1	Coaction	Attentive	A	Collaborative	Emotional Stroop	Nt	250	Mixed	Yes	Keyboard	2	30	Yes
Zhang et al. (2018)	3	48	No	21	100	W	1	Coaction	Attentive	A	Collaborative	Emotional Stroop	Nt	250	Mixed	Yes	Keyboard	3	30	Yes
Figueira & Garcia-Marques (2019)	1	48	Yes	22	100	B		Other			Incidental	Classic & Semantic	Nt	100	Separated	No	Keyboard	4	300	Yes
Figueira & Garcia-Marques (2019)	2	58	Yes	23	74	B	5-8	Coaction	Inattentive	I	Incidental	Classic & Semantic	Nt	100	Separated	No	Keyboard	4	300	Yes
Fernandes et al. (2019)	1	62	Paid	21	60	B	3-7	Coaction	Inattentive	I	Incidental	Emotional Stroop	Cg	700	Mixed	No	Keyboard	2	256	No
Spatola et al. (2019)	1	118	No	19	93	B	1	Bystanding	Attentive	A	Evaluative	Classic & Semantic	Nt	1000	Mixed	Yes	Keyboard	4	268	Yes
Bouhours et al. (2020)	1	101	No	17	56	B		Bystanding	Attentive	A	Evaluative	Classic & EmoStroop	Cg	1000	Mixed	Yes	Keyboard	3	144	
Bouhours et al. (2020)	2	90	No	17	53	B		Bystanding	Attentive	A	Evaluative	Classic & EmoStroop	Cg	1000	Mixed	Yes	Keyboard	3	144	
Garcia-Marques et al. (2020)	1	81	Paid	20	85	B	4-5	Coaction	Inattentive	I	Incidental	Classic & Semantic	Nt	500	xed/Separa	No	Keyboard	4	336	No
Fernandes et al. (2020)	1	76	Yes	21	89	B	4-5	Coaction	Inattentive	I	Incidental	Classic & Semantic	Nt	500	Separated	No	Keyboard	4	336	No

Legend. Exp. = Experiment number; N = Sample size; Credit = Course credit or payment; Age = Mean sample age; Sex = Percentage of females; Design = Between-subjects (B) or Within-subjects (W); NOth = Number of others in the presence condition; Context = Type of presence social context; Attention = Attentive others; Aa = Author's categorization of attention; Evaluation = Potential evaluation context from others; Type = Stroop task type; Control = Control trials, Neutral (Nt) or Congruent (Cg); RSI = Response stimulus interval (ms); Block = Presentation of incongruent and control trials; Time = Restriction of time to give a response; Response = Type of response; Choice = Number of response alternatives; Trials = Number of experimental trials; Pratice = Inclusion of training trials.

2. Data Extraction and Computation of Effect Sizes

The effect size metric used for both outcome measures (i.e., reaction times and error rates) was *Hedge's g* (H_g). For both outcomes, a positive value in H_g indicates a lower Stroop interference value in the presence of other conditions relative to the alone condition. We chose this metric instead of *Cohen's d*, which tends to overestimate the true effect size in small samples, especially those smaller than 10 participants (Borenstein, 2009; Field & Gillett, 2010); sample sizes reported in our dataset (*k*=5). We first estimated *g* as the standardized difference of means (Hedges & Olkin, 1985):

$$g = \frac{\bar{Y}_e - \bar{Y}_c}{s_p}$$

where $\bar{Y}_e$ is the mean of the interference index of the experimental condition (i.e., presence of others), $\bar{Y}_c$ is the mean of the control condition (i.e., alone), and s_p is the pooled sample standard deviation:

$$s_p = \sqrt{\frac{(n_e - 1)s_e^2 + (n_c - 1)s_c^2}{n_e + n_c - 2}}$$

Then, we multiplied *g* by the correction factor for the sample size J_m to compute H_g:

$$J_m = 1 - \frac{3}{4(n_e + n_c) - 1}$$

The advantage of the H_g metric is that it can also be computed from appropriate *t*, *p,* and *F* statistical values, which are predominantly reported in the studies included in this meta-analysis. Effect sizes were estimated directly from *t* statistics (or *F* statistics reporting specific contrasts) using the formula described by Borenstein (2009).

In some cases, we use the *t* (or *F* value) of a specific contrast reported in studies with *df* greater than 1 (or 1 in the case of 2 x 2 interactions) to derive the *MSE* of the specific factor and compute the contrasts of interest according to the *MSL* method, following the formulas indicated by Winer,

Brown, and Michels (1991). In cases where the *F* statistic of the factor of interest was reported, we computed the S_p by reconstructing the relevant terms of the analysis of variance (Winer et al., 1991; see also Johnson & Eagly, 2000), and if necessary, we proceeded to compute the contrasts of interest for *df* greater than 1 using the *MSL* method.

In cases where the *F* statistic of interest in multifactorial studies was reported, we reconstituted (whenever possible) the error term considering the variance of irrelevant factors to correct the effect size (*see* Johnson & Eagly, 2000; Morris & DeShon, 1997). However, this procedure had a negligible impact on the results of the meta-analysis.

For several cases, the factor of interest was the interaction between the *between-subjects* factor of the presence type (i.e., alone vs. presence of others) and the *within-subjects* factor of the trial type (i.e., incongruent vs. control). In a few studies, the presence of others was also manipulated *within subjects* (k = 8, of which four studies reported *F* statistics). In this case, we compute the effect sizes using the appropriate formulas for this type of design (*see Supplemental Materials*).

For the computation of within-subjects effect sizes, it is necessary to correlate with the experimental and control conditions, something rarely reported. Thus, we follow the common practice of assigning a correlation of .5 to these cases (Borenstein, Hedges, Higgins, & Rothstein, 2009) in the meta-analytical analyses presented here. Additionally, we performed meta-analytic modeling with five different levels of within-study correlations (0.1, 0.3, 0.5, 0.7, and 0.9) described in the *Supplemental Materials*, although we did not observe significance in the data pattern.

Additionally, for the 74 effect sizes included in the different meta-analyses (for both outcomes), we calculated the *Hg* variance to proceed with the meta-analytical analysis; the individual study's effect sizes were weighted by the inverse of its variance, resulting in larger weights for more precise effect sizes (Hedges & Olkin, 1985; Hedges & Vevea, 1998). The variance formula is the following (Borenstein, 2009):

$$V_g = J^2 \times \left(\frac{n_e + n_c}{n_e n_c} + \frac{d^2}{2(n_e + n_c)} \right)$$

3. Meta-analytic Procedures

Statistical analysis. All meta-analyses and publication bias analyses (for bias and error-rate outcomes) were performed using the *Metafor package* (*Version 3.2.0*, Viechtbauer, 2010) for *R* (R Core Team, 2008). *Hedges' g* (*Hg*) was used as an effect size metric and is a corrected version of *Cohen's d* that follows the same adopted conventions (Cohen, 1988), with the effect sizes interpreted as small (*Hg* > 0.2), medium (*Hg* > 0.5), and large (*Hg* > 0.8).

The meta-analysis follows a random-effects, multilevel approach (e.g., Raudenbush & Bryk, 2002). This multilevel approach allowed us to cope with any dependencies among the studied effect sizes (see Konstantopoulos, 2011; Viechtbauer, Kotz, Spigt, Arts, & Crutzen, 2014). We considered two levels of analysis, study, and paper because most papers in our dataset reported more than one study, and some studies contributed more than one effect size (associated with different levels of the moderators). The random effects of studies and papers were specified as a list of one-sided formulas in the random argument of the *rma.mv* function of *Metafor*. The *restricted maximum-likelihood* (*REML*) method was used, and the effect sizes with the same level within each grouping variable received the same random effect; otherwise, effect sizes were assumed to be independent (Viechtbauer, 2010).

We assessed heterogeneity across effect sizes (*see Table 1*) by calculating a 95% confidence interval (*CI*), *Cochrane's Q*, and I^2 statistics indicators. Parameter estimates were obtained via *REML,* and statistical tests of model coefficients were computed via *Wald-type chi-squared tests*. Heterogeneity between the included studies is indicated by a significant statistical test for *Cochrane's Q* (at the .05 level), and I^2 represents the proportion of variation due to heterogeneity concerning chance (Higgins & Thompson, 2002). If high heterogeneity between studies is verified for their outcomes, the existence of moderating variables is expected, which could be subsequently tested.

Categorical moderation and meta-regression. We also ran separate meta-analytic studies for each of the moderators associated with the presence of other manipulations. We analyze the categorical moderators specified in the meta-analytical model through the *mods* argument (*rma.mv function*), excluding the intercept. As the moderation test associated with *Cochrane's Q* statistic for these models is relative to zero, we computed the contrasts of interest between the moderator levels. This procedure is equivalent to doing *wald-type linear combinations* in a *one-way ANOVA* procedure. A significant effect means that between-class variance differs from the expected variance of the sampling error (Hedges & Olkin, 1985); that is, the moderator alters the effect of the presence of others on the outcome (in the case of binary moderators or between levels of moderators with 3 or more levels).

For the test of multiple factors (moderators) and their interaction, we started by dummy-coding the moderators of interest (i.e., social context and attention), allowing the combination of different levels of these factors. Then, we fitted a mixed-effects meta-regression model containing the main effects of the 2 factors (mods = Factor 1 + Factor 2) and a model for the interaction between the factors (mods = Factor 1 x Factor 2). Additionally, we parameterized the model (mods = Factor 1: Factor 2) to calculate the effect of each cell of the two-way ANOVA and proceeded to the calculation of the contrasts of interest.

A meta-regression procedure was used for continuous moderators based on a mixed-effects regression model.

Publication bias. Publication bias represents the potential tendency for researchers to report (and publish) statistically significant results, which can result in stronger effect sizes in the meta-analysis (Thornton & Lee, 2000). To check the presence of potential publication bias, we drew and analyzed for both outcomes, bias, and error rate), the corresponding funnel plots, which show the observed effect sizes as a function of its precision (i.e., 1/standard error). An asymmetry in this distribution indicates potential publication bias and was assessed with the *rank correlation test* as

described by Begg and Mazumdar (1994). A positive correlation indicates that there is an asymmetry in the funnel plot. Next, we applied the *trim-and-fill method*, which allows us to correct any potential publication bias. This method consists of trimming the outliers of the distribution and filling in hypothetical effects so that the funnel plot becomes symmetrical (Duval & Tweedie, 2000), and its result reflects the corrected overall effect and whether it remains statistically significant.
